# Functional characteristics of novel pancreatic Pax6 regulatory elements

**DOI:** 10.1093/hmg/ddy255

**Published:** 2018-08-10

**Authors:** Adam Buckle, Ryu-suke Nozawa, Dirk A Kleinjan, Nick Gilbert

**Affiliations:** 1 MRC Human Genetics Unit, Institute of Genetics and Molecular Medicine, University of Edinburgh, Edinburgh EH4 2XR, UK; 2 Centre for Mammalian Synthetic Biology, University of Edinburgh, Edinburgh EH9 3FF, UK

## Abstract

Complex diseases, such as diabetes, are influenced by comprehensive transcriptional networks. Genome-wide association studies have revealed that variants located in regulatory elements for pancreatic transcription factors are linked to diabetes, including those functionally linked to the paired box transcription factor Pax6. *Pax6* deletions in adult mice cause rapid onset of classic diabetes, but the full spectrum of pancreatic *Pax6* regulators is unknown. Using a regulatory element discovery approach, we identified two novel *Pax6* pancreatic *cis*-regulatory elements in a poorly characterized regulatory desert. Both new elements, Pax6 pancreas *cis*-regulatory element 3 (PE3) and PE4, are located 50 and 100 kb upstream and interact with different parts of the Pax6 promoter and nearby non-coding RNAs. They drive expression in the developing pancreas and brain and code for multiple pancreas-related transcription factor-binding sites. PE3 binds CCCTC-binding factor (CTCF) and is marked by stem cell identity markers in embryonic stem cells, whilst a common variant located in the PE4 element affects binding of Pax4, a known pancreatic regulator, altering *Pax6* gene expression. To determine the ability of these elements to regulate gene expression, synthetic transcriptional activators and repressors were targeted to PE3 and PE4, modulating *Pax6* gene expression, as well as influencing neighbouring genes and long non-coding RNAs, implicating the *Pax6* locus in pancreas function and diabetes.

## Introduction

Distal *cis*-regulatory elements are a major component of the mechanism asserting temporal and spatial patterns of gene expression. Understanding the function of regulatory elements has taken on new significance as they are increasingly linked to human phenotypic variation and complex disease phenotypes. However, as approximately 90% of disease-associated risk alleles fall within non-coding regions, a major challenge is pinpointing target genes and understanding underlying mechanisms of dysregulation (1).

Pax6 is an evolutionarily conserved pleiotropic transcription factor with roles in the development of the central nervous system (CNS), the eye and the olfactory system and is also critical for pancreas development and hormone production from endocrine secretory cells (2,3). In humans the congenital eye malformation aniridia is characterized by haploinsufficiency for the PAX6 protein, and studying this condition has enabled the identification of large *cis*-regulatory regions controlling *Pax6* expression (4). In the pancreas, multiple complex transcriptional networks utilize homeo- and paired-domain-containing transcription factors (such as Nkx2.2, Nkx6.1, Pdx1, Pax4, Isl1 and Pax6) (5) which are vital for coordinating the differentiation of progenitors to mature pancreatic cells (6) and directly regulate many pancreatic target genes (5,7). Pax4 is another well-studied pancreatic transcription factor and is expressed early in pancreas development, where it is essential for specification and maintenance, as mice null for *Pax4* have a severe diabetic phenotype (8). Pax4 is also important for adult β-cell function and is linked to human pancreatic disease; mutations and common risk alleles in *Pax4* have been linked to type 1 diabetes (T1D) and type 2 diabetes (T2D) (9–11).

Diabetes is caused by loss or dysfunction of the insulin-secreting pancreatic β-cells, with autoimmune loss causing T1D, whilst in T2D, insulin secretion is defective, which in turn brings about an imbalance in glucose homeostasis and insulin resistance (9). Pax6 is required for embryonic stem (ES) cell differentiation to neural lineages consistent with its critical role in neural development. It is also expressed in the early pancreatic bud and is necessary for insulin homeostasis in the adult pancreas (12,13); Pax6 deletion rapidly leads to classical diabetes and weight loss (13). A common regulatory variant (rs11603334G>A) for fasting pro-insulin levels (14,15) is located in the *ARAP1* promoter, a regulator of *PAX6*, whilst a genome-wide association with body mass index (*P* ≤ 5.0 × 10^−7^) is found upstream of the *PAX6* gene (16). Consistently a study of aniridia patients with heterozygous *PAX6* mutation found glucose intolerance characterized by impaired insulin secretion in all patients, demonstrating that the endocrine pancreas is sensitive to levels of PAX6 (17). Similarly genome-wide profiling of *cis*-regulatory networks in islet cells have shown an enrichment of T2D single-nucleotide polymorphisms (SNPs) in islet-specific enhancers, which themselves bind islet transcription factors (18).

**Figure 1:**
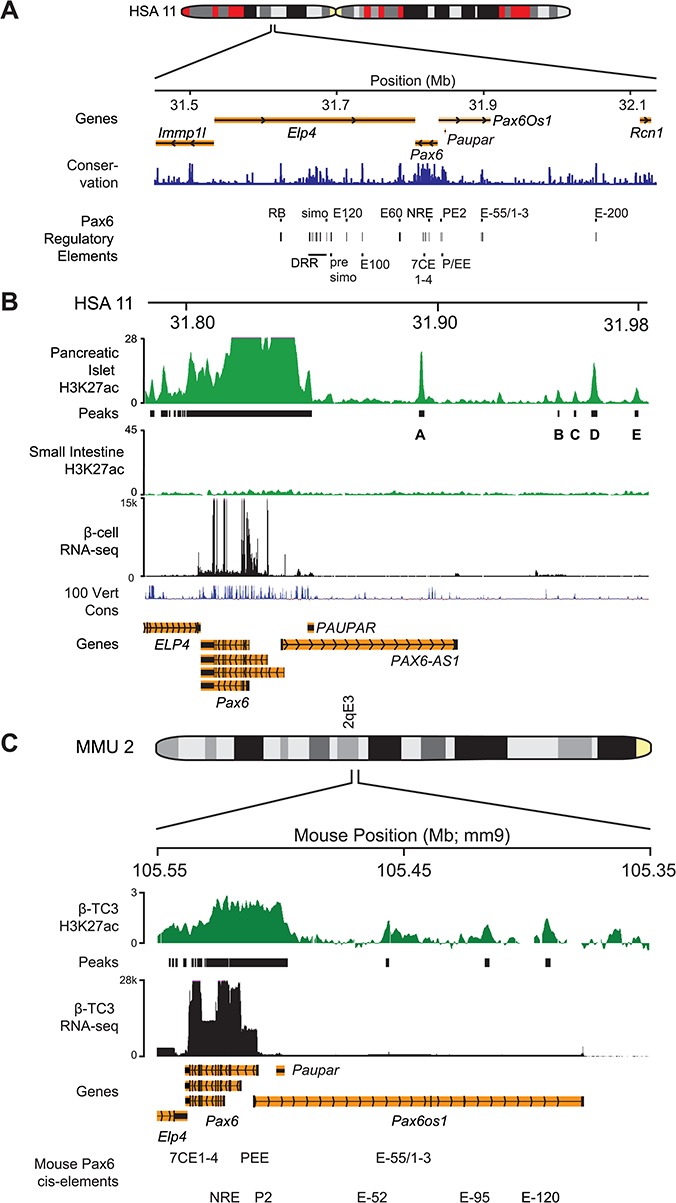
H3K27ac profiling identifies regulatory elements in the PAX6 locus. (**A**) Diagram showing the human *PAX6* regulatory domain with flanking genes. Genomic position of known *PAX6* regulatory elements are marked in black (UCSC liftover from mouse coordinates) and sequence conservation (blue). (**B**) Human *PAX6* locus with ChIP-seq for H3K27ac histone modification (green) in primary pancreatic islet and small intestine tissue from Human Epigenome Atlas, with MACS identified peaks (63). RNA-seq feature counts (black) for representative human β-cell sample (25) (Supplementary Material Table S1) and vertebrate conservation (blue). (**C**) ChIP-chip for H3K27ac histone modification in mouse β-TC3 cells (green), with called peaks. RNA-seq feature counts (black) for combined β-TC3 cell replicates. UCSC RefSeq genes (orange) and known Pax6 regulatory elements. ChIP data represents average (*n* = 2). hg19 and mm9 coordinates.

Within the 11p13 locus, the most critical gene encodes PAX6, which has a large regulatory domain with multiple long-range elements, many of which reside within introns of its neighbouring gene, *ELP4*, including the downstream regulatory region, a complex enhancer cluster of tissue-specific hypersensitive sites (19) (Fig. 1A). Of importance for pancreatic function, the pancreas and ectoderm enhancer (P/EE) cluster drives α- and β-cell-specific expression during development and after birth (20), and a pancreas-specific regulatory element [Pax6 pancreas *cis*-regulatory element 2 (PE2)] drives stable endocrine pancreas expression during development and into adulthood (21). Targeted deletions of P/EE and PE2 regions reduce *Pax6* expression in the pancreas (22) but do not abolish it; we therefore hypothesized that other, as yet unidentified, pancreas-specific *Pax6* regulatory elements exist and would be functionally conserved between human and mouse and reveal novel insight into *Pax6* pancreatic function and regulation.

To identify and functionally characterize novel regulatory elements in the *PAX6* regulatory domain, we analysed human pancreatic tissues, mouse pancreatic β-cells (β-TC3) and ES cells as tractable experimental systems. Two elements were identified that associated with chromatin marks indicative of regulatory function in human and mouse pancreatic cells; called PE3 and PE4, they acted as regulatory elements in mouse reporter transgenics, revealing a neural and pancreatic expression pattern. Functional characterization of PE3 showed it was bound by CCCTC-binding factor (CTCF) and engaged the *Pax6* gene via regulatory looping over a 50 kb region, in both pancreatic cells and in mouse embryonic stem cells (mESCs), whilst PE4 showed a more complex interaction profile in pancreatic cells interacting with PE3, the *Paupar* long non-coding RNA (lncRNA) and the *Pax6* gene. Within the PE4 element, a common variant, rs7943160G>C, is positioned in a vertebrate conserved PAX4-binding motif and alters reporter expression in a PAX4-dependent manner, linking two pancreatic transcription factors. Finally, we demonstrated the importance of these *cis*-regulatory sites by recruiting transcription activator-like (TAL) effectors fused to transactivator or repressor domains to the PE3 and PE4 elements to modulate the expression of Pax6 and surrounding genes.

## Results

### Identification of novel human and mouse conserved pancreatic regulatory elements

In contrast to the downstream region of *PAX6*, which has a significant role in disease aetiology of aniridia patients, the upstream region towards *RCN1* is less well studied; it includes the only E-200 element and E-55 cluster, implicating this region as a regulatory ‘desert’ even though it is known to contain a number of evolutionarily conserved sites (23,24). As *cis*-regulatory elements are key to modulation of gene expression through transcription factor binding and are increasingly linked to complex disease phenotypes, we set out to identify and characterize novel *PAX6* regulatory elements specific to pancreatic expression, owing to its roles in pancreatic development and T2D. Reasoning that novel regulatory elements would be marked by enhancer-specific histone modifications, we mined histone H3K27ac and H3K4me1 chromatin immunoprecipitation (ChIP) data sets from human primary pancreatic islet tissue (Human Epigenome Atlas). Analysis of the region upstream of the *PAX6* gene suggested it might harbour a number of putative *PAX6* regulatory elements. Peak calling was used to identify pronounced ChIP signal enrichment, marking five discrete peaks in the upstream region (Fig. 1B), labelled A–E, as putative novel *cis*-regulatory elements. Analysis of transcriptome data from primary purified human pancreatic beta cells (25) confirmed high *PAX6* expression, but unexpectedly two lncRNAs, *PAUPAR* and *PAX6-AS1*, located upstream of Pax6 were also expressed in β-cells (Fig. 1B). Quantification across six individual primary β-cells (Supplementary Material Table S1) indicated that *PAUPAR* was consistently expressed [mean fragments per kilobase million (FPKM) 5.7] and aligned with the islet H3K27ac signal. The second lncRNA *PAX6*-AS1 is analogous to mouse *Pax6OS1* (26) (mean FPKM 10.2), and together this expression data suggests that *PAX6* locus lncRNAs may have a role in pancreatic cells.

Although this data hinted at the presence of regulatory elements, H3K27ac enrichment alone is not definitive for their identification. Important regulatory elements are likely to be well conserved in sequence and function between mammalian species, so putative regulatory marks in mouse cells were investigated. The strict tissue specificity of *Pax6* expression requires that ChIP enhancer profiling must be performed in a suitable cell type to identify appropriate active regulatory elements. Few pancreatic β-cell lines are available, but mouse pancreatic β-TC3 cells are used in many studies and express *Pax6* at a high level (27), so these provide a suitable model. ChIP-on-chip for the active enhancer modification H3K27ac was performed and mapped across the *Pax6* region using custom tiling arrays in β-TC3 cells (Fig. 1C). The H3K27ac modification marked the *Pax6* promoter region and promoters of the adjacent ubiquitously expressed genes, *Elp4, Immpl1* and *Rcn1*. H3K27ac enrichment extended beyond the *Pax6* promoters, upstream where known *Pax6* pancreatic elements P/EE and PE2 are located and over the *Pax6* intron 7 enhancer cluster, 7CE1-4. Strikingly the mouse β-TC3 H3K27ac signal showed multiple novel H3K27ac-enriched regions in the *Pax6* upstream region (labelled elements E-52, E-95 and E-120; 52, 95 and 120 kb 5′ from the mouse P0 promoter, respectively). There was high concordance between the β-TC3 and the human pancreatic islet data; human H3K27ac peaks A and B share core sequences with the mouse H3K27ac peaks E-52 and E-120. Previously, only the P/EE and PE2 elements were characterized as PAX6 pancreatic enhancers (20–22); thus, identification of these new elements significantly expands the repertoire of regulatory regions with a potential role in controlling *PAX6* expression in the pancreas. High-resolution analysis of mouse and human DNase I ENCODE data confirmed that these elements were discrete (data not shown) and so were named as putative *cis*-regulatory pancreatic element 3 (PE3; corresponding to human peak A and mouse E-52) and PE4 (corresponding to human peak B and mouse E-120) (Fig. 1).

**Figure 2:**
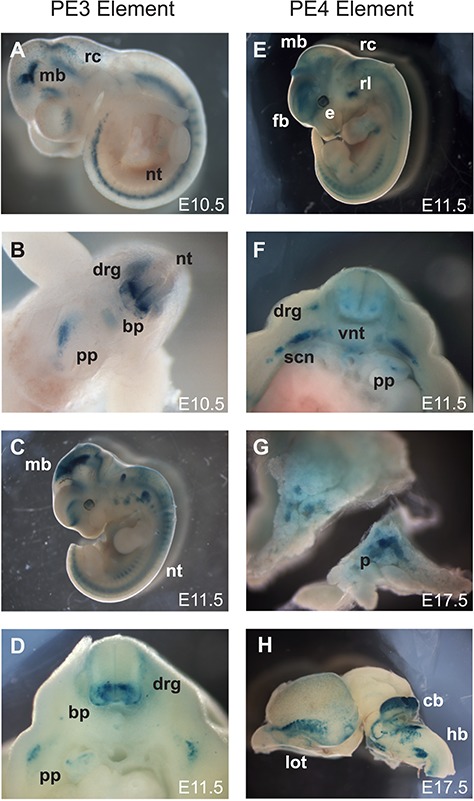
PE3 and PE4 *cis*-regulatory elements drive neural and pancreatic expression in mouse embryos. Stable mouse line with PE3 reporters showed strong signal in *Pax6* expressing tissues at E10.5 (**A**, **B**) and E11.5 (**C**, **D**), whilst PE4 showed a more restricted *Pax6* expression pattern at E11.5 (**E**, **F**) and E17.5 (**G**, **H**). (**A**) Lateral view of E10.5 embryo showed LacZ staining in the ventral cerebral vesicles (cv) of the telencephalon, the ventral midbrain (mb), rhombencephalon (rc) and neural tube (nt). (**B**) Transverse section through body of E10.5 embryo showed ventral (nt) staining in neurogenic region of the basal plate (bp), dorsal root ganglion (drg) and visible staining in the pancreas primordium (pp). (**C**) Lateral view of PE3 embryo at E11.5 showed ventral forebrain expression (vf), staining in (mb), the (rc) into the (nt) and the developing eye (e). (**D**) Transverse section through the body of E11.5 embryo revealed strong ventral (nt) staining in the neurogenic region of the basal plate (bp) of the (nt), plus the dorsal root ganglion (drg) surrounding the (nt), and (pp) in the body cavity. (**E**) Lateral view of E11.5 embryo with staining in the forebrain (fb), ventral (mb), rhombic lip (rl) of rhombencephalon (rc), neural tube (nt) and (e). (**F**) Transverse section through the body showed diffuse ventral neural tube (vnt) staining, dorsal root ganglion (drg), sympathetic chain neurons (scn) below the neural tube and (pp) staining in the body cavity. (**G**) Dissected E17.5 pancreas (p) cut into two cross sections revealed internal staining pattern. (**H**) Lateral view dissected E17.5 brain showed nerve tracts in the forebrain consisting of lateral olfactory tracts (lot) and ventral brain nuclei staining in cerebellum (cb) and hindbrain (hb).

We hypothesized that gene expression patterns as well as *cis*-regulatory elements would be conserved between mouse and human pancreatic cells, so RNA-seq in β-TC3 cells was performed (Fig. 1B). This revealed high *Pax6* (mean FPKM 110) and *Paupar* and *Pax6Os1* lncRNA (mean FPKM 2.2 and 6.2) expression (Supplementary Material Table S1), as seen in human pancreatic samples. To further assess the cell type specificity of these putative elements, we analysed Epigenome Atlas H3K4me1 ChIP-seq data across 48 distinct human tissues (Supplementary Material Fig. S1). There was visible enrichment of H3K4me1 signal over the PE3 region in 45% of tissues assayed, whilst PE4 only showed enrichment in pancreatic islet tissue samples (PI_13 and PI_27), suggesting it was pancreas specific.

### PE3 and PE4 elements transactivate expression in mouse reporter transgenics

To assess the spatio-temporal characteristics of the novel PE3 and PE4 regulatory elements, we generated LacZ reporter transgenic mice (20,21). The putative regulatory elements were cloned into an *Hsp*68 minimal promoter–LacZ reporter construct (4) and used to generate transient transgenic embryos and stable lines that were analysed at different developmental stages (Supplementary Material Fig. S2, Fig. 2). For the PE3 element, six independent E11.5 dpc transient transgenic embryos were analysed before obtaining two stable lines, one of which was studied in detail across multiple developmental stages. Consistent staining between transient embryos and stable lines was observed in regions of the pancreas, brain and neural tube (Fig. 2A–D). Midbrain expression was consistently observed for the PE3 element which is not a site of *Pax6* expression; however, such occurrences of ectopic expression sites are not unusual in reporter transgenics when the element is not in its correct genomic context (28).

At E10.5 the PE3 element showed staining in the pancreas primordium, parts of the CNS including along the neural tube in the basal plate and dorsal root ganglia and the ventral midbrain and hindbrain (Fig. 2A and B). At E11.5 staining was visible in the midbrain and hindbrain, whilst opening up the body cavity revealed further pancreatic staining (Fig. 2C and D). During later stages of development, expression became restricted to specific regions of the CNS, in particular the lateral olfactory tracts, midbrain, cerebellum and regions of the hindbrain (Supplementary Material Fig. S2A and B).

For the PE4 element, three stable lines were obtained and consistent expression was observed in a more restricted manner in the pancreas and specific regions of the developing brain. The PE4 element showed a more diffuse staining pattern in the CNS at E11.5 (Fig. 2E), but some staining could be seen in the centre of the pancreas primordium (Fig. 2F). At E17.5 the pancreas showed strong staining in islet-like cells (Fig. 2G), consistent with the reported *Pax6* expression pattern (29). Also, at E17.5 the lateral olfactory tract, cerebellum and focal regions of the hindbrain showed expression (Fig. 2H and C).

These results support PE3 and PE4 to be novel tissue-specific regulators driving reporter expression in embryonic pancreas and neuronal tissues. PE3 has a broad *Pax6* expression pattern across multiple stages of development, whilst PE4 is more restricted to the developing pancreas.

### Novel *Pax6* regulatory elements show sequence conservation, transcription factor binding and encode putative regulatory SNPs

**Figure 3:**
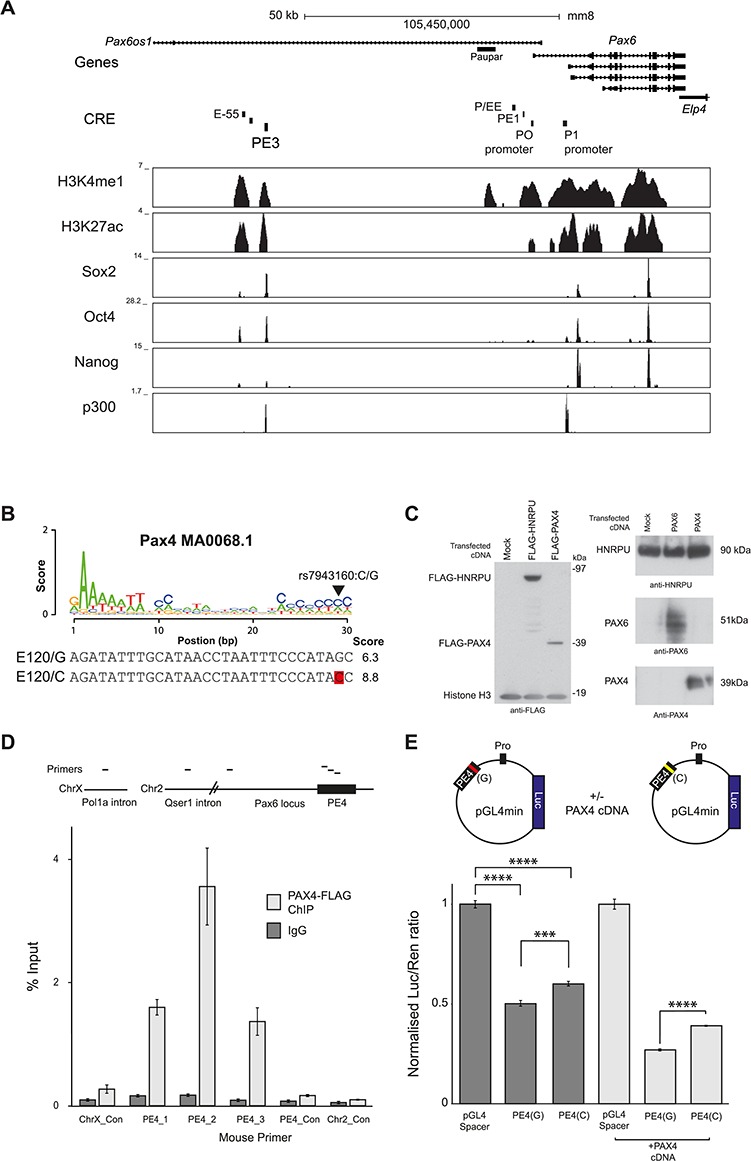
Histone modification and transcription factor binding across PE3 at the *Pax6* locus in mESC and characterization of a PE4 regulatory variant. (**A**) 100 kb genomic region surrounding the PE3 element at the *Pax6* gene showing histone modifications and transcription factor binding in mESC (32). (**B**) Position of putative regulatory SNP in Pax4 binding sequence, with motif and alignment score from Jaspar database (64). (**C**) (left) Western blot analysis of β-TC3 cells transfected with FLAG-PAX4 or FLAG-hnRNPU with a mock transfection control, detected with an antibody against FLAG and using histone H3 antibody as a loading control. (right) Western blot analysis of β-TC3 cells transfected with untagged PAX6 or PAX4 cDNAs and a mock transfection control, probed with antibodies against PAX4, PAX6 and hnRNPU as a loading control. (**D**) ChIP assay for FLAG-PAX4 in β-TC3 cells, evaluated by qPCR, using primer sets to the mouse PE4 element. Top, schematic showing primer locations, with negative control primers to a sequence 4 kb upstream of PE4 (PE4_Con), intergenic regions on Chr2 (Chr2_con) and a large intron in Pola1 on HSAX (chrX_Con). Values are displayed as percentage of input sample, with two independent experimental replicates of FLAG-PAX4 ChIP. Error bars ±SEM. (**E**) Dual luciferase assay for human PE4 element encoding the G or C variant in pGL4.23 vector, transfected into β-TC3 cells, co-transfected with human PAX4 cDNA. Relative luciferase signal represents firefly luciferase over *Renilla* luciferase signal, normalized to the signal from the empty vector. Error bars ±SEM, *n* = 5; *P*-value from Welch’s two-sample *t*-test (*P*-values: ^****^ <0.0001, ^***^<0.001, ^**^<0.01, ^*^<0.05).

To directly compare between the human and mouse loci, sequence conservation at the putative PE3 and PE4 elements along with conserved transcription factor-binding motifs within their sequences was examined to identify pathways and potential regulators. The PE3 element contained a 474 bp block with 58% sequence identity between human and mouse (Supplementary Material Fig. S3A), with a fully conserved 38 bp core across multiple mammalian species (data not shown). Transcription factor-binding motifs were identified within the element for the Sox and Oct genes (30). Gene expression analysis of these transcription factors using the Human Protein Atlas (31) revealed that nine were expressed (FPKM > 1) in adult pancreas, suggesting they are candidate transcription factors for binding to this element (Supplementary Material Table S2). Based on identification of pluripotency motifs, we next investigated published ChIP-seq data over the *Pax6* region in mESCs and noted that the PE3 element and neighbouring E-55 elements were associated with active H3K27ac and H3K4me1 enhancer modifications, the p300 transcriptional co-activator and pluripotent transcription factors Sox2, Oct4 and Nanog (Fig. 3A) (32). Sox2 and Oct4 have been shown to bind the *Pax6* promoter in mESCs and displace nucleosomes over the region (33). Consistently, *Pax6* is one of a group of transcription factors which are bivalently marked in embryonic stem cells (ESCs) by H3K4me3/H3k27me3 ready for activation upon differentiation (34). In addition to these pluripotency factors, sequence analysis using the CTCF Binding Site Database prediction tool (35) identified a conserved CTCF binding site in PE3 (Supplementary Material Fig. S3A), and ChIP in mouse β-TC3 cells confirmed this site binds CTCF *in vivo* (Supplementary Material Fig. S3B). Together this data revealed PE3 is a novel distal site of active transcriptional regulation of *Pax6* in mESCs, which binds important pluripotency transcription factors and is occupied by a key regulator of 3D chromatin organization (36).

Alignment of human and mouse sequences for the PE4 element identified a 802 bp fragment with 74% sequence identity between human and mouse (Supplementary Material Fig. S3C), containing multiple conserved transcription factor-binding motifs (Supplementary Material Table S3), including motifs for the important pancreas developmental regulators Nkx2.2 and Pax4 (8,37). Furthermore, gene expression analysis showed that 19 out of 28 transcription factors with putative recognition motifs in the element are expressed in adult pancreas (Supplementary Material Table S3) and indicate that PE4 is a more pancreas-specific element than PE3.

*Cis*-regulatory elements that are expected to have a major role in common disease phenotypes are likely to harbour common variants within the population that could modulate their regulatory activity. To address whether any of the common SNPs found in PE3 and PE4 could disrupt transcription factor binding, we scanned the human elements for SNPs embedded within transcription factor motifs. PE3 harbours the two SNPs rs11031498 and rs11031499, but these did not coincide with any transcription factor-binding sites. In contrast, rs7943160G>C within PE4 is a common variant in the population (Supplementary Material Fig. S4) which overlapped three transcription factor-binding motifs: KAISO, MZF1 and Pax4 (Supplementary Material Table S3).

### A common variant in PE4 alters regulatory element reporter activity

Pax4, an important regulator of pancreatic development, is expressed in adult α- and β-islet cells (10). As PAX4 has been linked to both T1D and T2D (9,11) and islet function, we investigated variants in its binding motif in PE4. SNP rs7943160 altered position 29 of the 30 bp PAX4 motif (MA0068.1) (Fig. 3B), with the motif having a stronger match for the ancestral C base over the common G variant. Multi-species sequence conservation analysis of PE4 revealed the PAX4 motif was seen across 59 mammals, with strong conservation of a C base in the aligned position of interest (94.8% of species) (Supplementary Material Fig. S5). This suggested that it is an important nucleotide position and thus a candidate for a common genetic variant that would alter PE4 regulatory function and *PAX6* expression. To evaluate PAX4 binding to PE4, β-TC3 cells were transfected with a FLAG peptide tagged version of human PAX4 (Fig. 3C); the FLAG-PAX4 ChIP signal over mouse PE4 was highly enriched compared with control regions (Fig. 3D), so it demonstrated that PAX4 can bind to the PE4 element.

As the PAX4 motif in PE4 contains a variant that may alter PAX4 binding affinity, the effect of the rs7943160G>C variant on the activity of the human PE4 element was assessed using a dual luciferase reporter assay in β-TC3 cells. The conserved region of the human PE4 element, with either the rs7943160 G or C variant, was cloned upstream of a minimal promoter driving luciferase (Fig. 3E) and transfected into β-TC3 cells. Both variants of the PE4 element showed a decrease in luciferase signal compared with the empty vector, demonstrating repressive behaviour. Importantly there was a striking and significant change in luciferase signal between the PE4(G) and PE4(C) variants (*P* < 0.001). To assess the effect of PAX4 on the variant binding sites, human *PAX4* cDNA was co-expressed with luciferase constructs in β-TC3 cells (Fig. 3E). As PAX4 is a transcriptional repressor of pancreatic genes (38), based on the predicted effect of the variants on the strength of the PAX4 motif *in silico* (Fig. 3A), we hypothesized that the G variant element would have lower binding affinity for PAX4, resulting in decreased repression and an increased luciferase signal. Both PE4 variants showed more repression when PAX4 cDNA was co-expressed and there was a significant difference between the two alleles (*P* < 0.0001), which was exaggerated compared with non-PAX4 cDNA-transfected samples (Fig. 3E). Together this indicated that a common regulatory variant in PE4 can alter regulatory element function and *PAX6* gene expression.

### Chromosome conformation capture reveals PE3 and PE4 regulatory looping to *Pax6*

**Figure 4:**
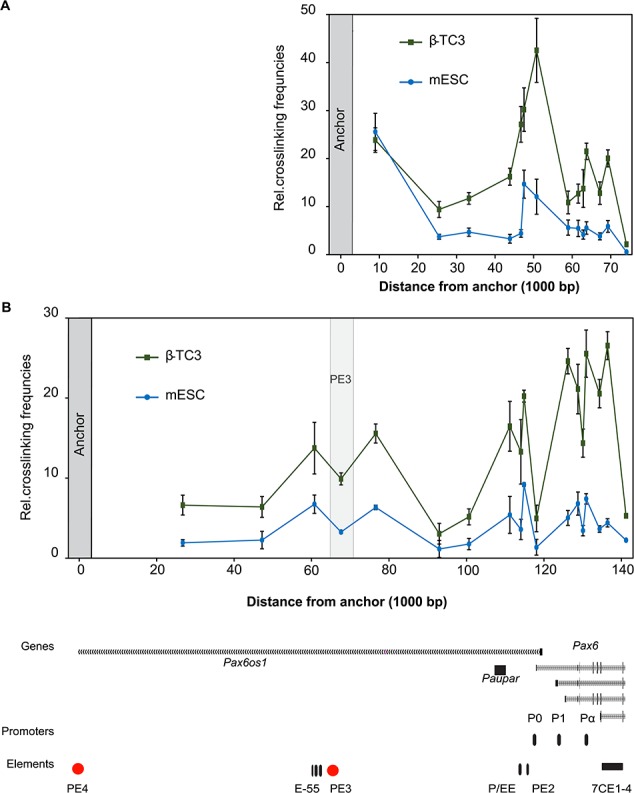
3C-qPCR reveals PE3 and PE4 interaction with multiple regions over *Pax6* gene. (**A**) PE3 3C-qPCR data displayed as relative cross-linking frequencies between the PE3 anchor fragment and *Bgl*II fragment primers across the mouse *Pax6* locus in β-TC3 and mESC. (**B**) PE4 3C-qPCR data from the PE4 anchor fragments in β-TC3 and mESCs. Data sets are scaled to one another and display genomic position relative to 3C primer values. Data from the two cell lines were normalized using a probe located in the *Ercc3* locus. The signal is the combined mean relative cross-linking frequency of six to eight individual qPCR values, across two experimental replicates. Error bars ±SEM.

Looping interactions have been proposed as a mechanism for regulatory elements located 10–100 kb from target sites to interact and influence gene activity. Previously, chromosome conformation capture (3C)-qPCR has been used to analyse interactions at multiple loci, so we selected this approach for characterizing regulatory interactions around *Pax6* from the PE3 and PE4 elements. We first investigated PE3 as it binds CTCF (Supplementary Material Fig. S3C), and CTCF’s role in coordinating gene regulatory looping is well established (36). PE3 was used as an anchor site from where relative interaction frequency was assayed at regular intervals across a panel of primers covering the 74 kb genomic landscape from PE3 to the *Pax6* gene. The PE3 element showed increased cross-linking frequency over the transcription start site (TSS) of the *Paupar* lncRNA, whilst the signal increased further at 47 kb away from the anchor, over a region of high H3K27ac upstream of the promoter (Fig. 1B), at the location of two known pancreatic regulatory elements P/EE and PE2, and then peaked over the *Pax6* P0 and P1 promoter fragments (Fig. 4A). This data showed that PE3 is a regulatory element which sits in spatial proximity to active *Pax6* promoters in β-TC3 cells. The binding of pluripotency transcription factors at both the PE3 enhancer and promoter may facilitate *Pax6* being maintained in a poised state (39). As such, we hypothesized that the PE3 element would be important for initial *Pax6* gene activation and may be involved in priming the gene for subsequent expression in specific embryonic lineages such as neuroectoderm. Consistently 3C-qPCR in mESCs revealed a more discrete interaction profile with signal peaking over background 3 kb upstream of the P0/P1 *Pax6* promoter, but reduced compared with that seen in β-TC3 cells (Fig. 4A).

We next hypothesized that PE4 would interact with the *Pax6* promoter and gene body and these interactions would be detectably higher than in mESCs where the element is not marked as active by H3K27ac. As predicted, β-TC3 cells showed a complex profile with substantially higher relative cross-linking at both PE3 and multiple regions of the *Pax6* gene promoters and gene body than in mESCs (Fig. 4B), though the mESC sample did show increased signal over the PE3 and *Pax6* gene, suggesting the whole locus may be in a conformation permissive for further activation. In both β-TC3 and mESC the relative cross linking frequency from PE4 was higher over a broad region encompassing the E-55 and PE3 elements (Fig. 4B), consistent with this region being H3K27ac positive in mESCs and β-TC3 cells (Figs 1B and 2A). Interesting the two elements showed very distinct interaction patterns to the Pax6 promoter, indicating that regulatory elements have a high degree of specificity for targeting associations between specific regulatory regions.

### Recruitment of synthetic transcription factors to PE3 and PE4 regulates gene transcription

**Figure 5:**
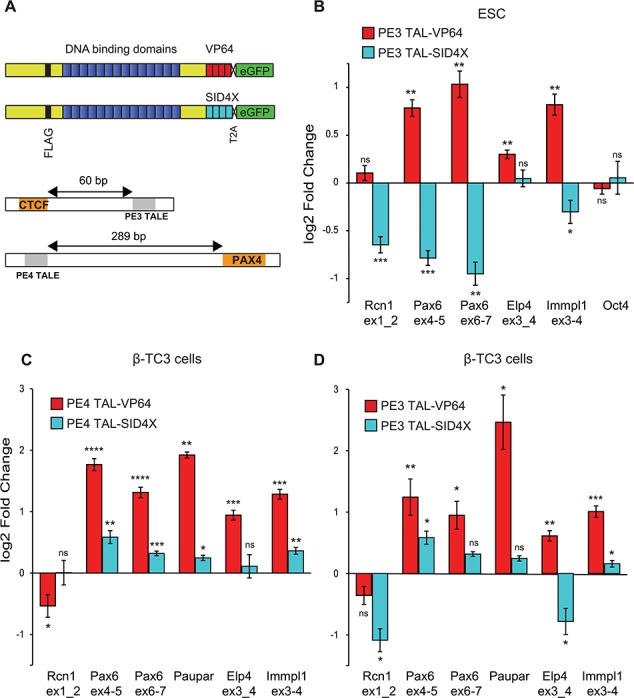
TALE recruitment to regulatory elements can modulate target gene expression. (**A**) Schematic showing TALE proteins designed to PE3 and PE4 regulatory elements fused to activator VP64 or repressor SID4X domains, with co-expressed GFP. Position of PE3 and PE4 TALEs with respect to CTCF and PAX4 binding sites. (**B**–**D**) Graph showing relative change in *Pax6*, *Paupar*, *Elp4, Immpl1*, *Rcn1* and *Oct4* expression after transfection with TALEs fused to VP64 or SID4X targeted to the PE3 or PE4 regulatory elements in β-TC3 or mESC. RNA levels were quantified by RT-PCR and normalized to 18S RNA. Error bars ±SEM, *n* = 2. *P*-value from a Welch’s *t*-test of ΔCT values (*P*-values: ^****^<0.0001, ^***^<0.001, ^**^<0.01, ^*^<0.05).

Characterization of *cis*-regulatory elements outside of the native genomic environment can only provide an incomplete picture of their function. TAL effectors (TALEs) are a class of proteins that have been used to modulate transcription, epigenetic states (40,41) and nuclear organization (42,43). As regulatory elements are landing pads for transcription factor binding, which transfer signals and factors onto gene promoter sequences, we reasoned that targeting a regulatory element with a transactivator could be a useful approach to understand its specificity and behaviour. To examine the ability of the PE3 and PE4 elements to influence native *Pax6* expression, we used a synthetic transcription factor modulation system (Fig. 5A) using TALEs targeted to *cis*-regulatory elements coupled to either a VP64 transcriptional co-activator (42) or a SID4X transcriptional repressor (40,44) (Supplementary Material Fig. S3A and C). We reasoned that transcriptional modulation by synthetic transcription factor recruitment at the distal regulatory sites would affect *Pax6* transcription if the elements were bona fide *cis*-regulatory elements for the gene.

As the PE3 element is a site for Sox2 and Oct4 binding (Fig. 3A), TALE constructs were tested in mESCs. Constructs were transfected into cells, enriched and RNA purified for qRT-PCR. TAL-VP64 targeted to PE3 caused a significant 1.6- to 2-fold increase in *Pax6* expression (Fig. 5B), whilst expression of the TAL-SID4X (Fig. 5A) repressor promoted a similar reduction in *Pax6* expression. The neighbouring genes *Elp4* and *Immpl1* (∼235 kb downstream from *Pax6* promoter) showed an increase in expression, whilst the upstream gene *Rcn1* (∼270 kb away) showed no significant change in expression, nor did the control *Oct4* gene (Fig. 5B)*.* In contrast, SID4X recruitment reduced expression of both the *Pax6* and *Rcn1 genes.*

To test the activity of the PE3 and PE4 elements in a pancreatic environment, constructs were expressed in β-TC3 cells. qRT-PCR for *Pax6* revealed significant transcriptional upregulation (2.5-to 3.4-fold) (Fig. 5C) after VP64 recruitment, demonstrating that PE4 can act over 120 kb to affect *Pax6* expression. As the H3K27ac signal in β-TC3 cells (Fig. 1B) extends ∼8 kb upstream of the P0 promoter over the lncRNA *Paupar* (45), we hypothesized that *Paupar* might be active in β-TC3 and modulated by factor recruitment to PE4. *Paupar* was indeed expressed to a similar level as *Pax6* and showed a strong and significant 4-fold increase in expression upon VP64 recruitment (Fig. 6B). As for mESCs, neighbouring genes were also influenced by VP64 recruitment, particularly downstream of the gene, consistent with promoters interacting locally with each other (46), but suggesting that *Rcn1* might be too far away to be strongly influenced by *Pax6* elements. SID4X recruitment did not have the repressive effect on gene expression seen in mESCs, but surprisingly induced a small increase in expression at *Pax6*, *Paupar* and *Immpl1*. VP64 recruitment to PE3 had an even greater effect on *Paupar* expression than PE4 activation (Fig. 5D), so we speculated that PE3 and PE4 function might be conserved in human pancreatic beta cells and might regulate *Paupar* lncRNA in a similar manner.

To further investigate the co-expression of Pax6 with surrounding genes, the correlation between gene expression across 23 RNA-seq samples in mouse tissues and cell lines (Supplementary Material Fig. S6A) was analysed. Pax6 expression was well correlated with downstream neighbouring genes, *Pax6Os1* (0.73) and *Elp4* (0.53), and showed little or a negative correlation with genes further upstream, *Rcn1* (0.004) and *Wt1* (−0.73). Consistently Pax6 is in a shared topologically associated domain (TAD) with surrounding genes as seen by HiC data visualization in mESCs (Supplementary Material Fig. S6B).

## Discussion

The *Pax6* locus has a densely packed regulatory landscape with more than 15 distinct regions of regulatory activity and many more predicted sites. Using complementary approaches, we identified two novel *Pax6* regulatory elements that are conserved in sequence and function between human and mouse. These novel elements drive diverse tissue-specific developmental expression patterns, at multiple stages of mouse development, and act as key signal input sites for gene expression modulation in pancreatic and ES cells.

Transcription factor binding is a critical property of regulatory elements; multiple putative binding sites including CTCF and Pax4 were identified and characterized at PE3 and PE4, respectively (Supplementary Material Fig. S3), along with components of the ESC transcription factor network, Sox2, Oct4 and Nanog (Fig. 2A). CTCF is often considered as a chromatin looping factor; consistent with this, we showed that PE3 interacted with the *Pax6* promoter region in mESCs. We propose that PE3 also has a role in recruiting the transcriptional apparatus to *Pax6* in differentiated β-TC3 pancreatic cells and may be an element important for general Pax6 gene activation in multiple tissues including the pancreas, consistent with its broad developmental expression pattern (Fig. 2). Recruitment of pre-initiation complex components to distal elements has been described at early stages of transcription at globin genes (47,48) and likely facilitates efficient transfer of factors ready for later differentiation. Using synthetic transcription factors, we confirmed that PE3 can transfer both activator and repressive signals over more than 45 kb to modulate *Pax6* gene expression. Interestingly we were able to repress *Pax6* gene expression in mESCs via TAL recruitment, but not β-TC3 cells using SID4X; this could be due to the mechanism of action of the Sin3 domain, which likely acts via Hdac1/2 and histone deacetylation (49). The correct combinations of factors may not be bound or available in β-TC3 cells or the factors involved in transcriptional activation may override these signals. ESC transcription may also be more plastic and more easily modulated; early establishment of enhancer activity and a poised bivalent gene expression state in ESCs is proposed as a means of efficient activation of target genes on differentiation, and bivalent marks are focused on homeodomain transcription factors such as Pax6 (39). This is supported by Sox2 binding to neural lineage-specific genes in advance of gene expression, which are then activated during differentiation (50). We propose that early activation via regulatory looping of PE3 (Fig. 4A) may transfer ESC transcription signals which can specify ESC identity (39) and likely poises *Pax6* for rapid activation as a transcription factor in neural differentiation.

The PE4 element has a more tissue restricted expression pattern than PE3 (Fig. 2), and in a pancreatic cell line model, PE4 was regulated by a well-characterized pancreatic regulator, PAX4, through direct binding to the element (Fig. 3). Furthermore, the human PE4 element encodes a single nucleotide common variant in a conserved PAX4 motif (Fig. 3B), which modulates its reporter activity in a PAX4-dependent manner. This demonstrates that a *PAX4*/*Pax6* regulatory network can be modulated by sequence variants found in the population (Supplementary Material Fig. S4). Both PAX4 and PAX6 have a well-established link to diabetes phenotypes (9,12,13,17). PAX4 is linked to the susceptibility of β-cells to apoptosis, leading to diabetes (9), and is found to be significantly differentially expressed within a T2D cohort of adult islets (10). Similarly, mutations in a number of transcription factors for islet function, including PAX4, PAX6 (3), HNF1 A (51), Nkx2.2 (52) and SOX4 (53), cause diabetes or diabetes-like phenotypes in human and mouse. Unsurprisingly genome-wide association studies for T2D are now revealing common variants at islet transcription factor loci (54). Pax6 regulates multiple β-cell-specific genes (insulin 1 and 2, *Pdx1*, *GLUT2*, Nkx6.1) (7), so subtle dysregulation of a master regulator may cascade through transcriptional networks to cause downstream phenotypic effects. Such a scenario fits with a study suggesting a link between sequence variation in pancreatic islet cell enhancers and T2D (18). Therefore, the identified functional variant in the PE4 element might play a role in modulating gene function and contribute to tissue-specific human phenotypic variation. As Pax6 expression in the adult pancreas has been shown to be important for islet maintenance and function, we propose a model where subtle variations in the tight regulation of Pax6, via a *cis*-regulatory mechanism, interact with other genetic and environmental risk factors to affect T2D disease risk.

Synthetic transcription factor recruitment to a *cis*-regulatory element is an important tool to test and functionally dissect element activity and specificity. Using synthetic transcription factors (Fig. 5), we showed that PE3 and PE4 could regulate *Pax6* expression in pancreatic β-TC3 cells. We also found that TAL recruitment influenced neighbouring gene expression to suggest a complex interplay between locally interacting or regulating genes. This could be directly through shared element interactions with neighbouring genes in the same TAD or via gene clustering in a hub influencing one another’s expression (55). This questions the idea of regulatory elements controlling single target genes and is consistent with experiments using the sleeping beauty transposon as a regulatory sensor, which shows that much of the genomic region around target genes are permissive to regulatory signals (56).

The *Pauper* lncRNA was upregulated by VP64 recruitment to PE3 and PE4 elements, this combined with interaction data that showed a high relative cross-linking frequency over the *Pauper* gene indicates that it is a target of these elements. *Pauper* was previously identified as a CNS-specific lncRNA expressed from the *Pax6* locus that was able to bind multiple regulatory elements in *cis* and *trans* and linked to intraocular tumours (45,57). Using short hairpin RNA-mediated downregulation of *Paupar,* Vance *et al.* (45) showed it could transcriptionally regulate *Pax6*, whilst *Pax6* knockdown did not affect *Paupar* levels, suggesting that the mechanism was not via a Pax6 autoregulatory affect. Our data indicates that *Pax6* and *Paupar* are coupled at the level of transcription, potentially via shared elements or linked promoter activity. In a recent study of human pancreatic β-cell lncRNAs, Akerman *et al.* (58) found that cell-type-specific lncRNAs play an important role in transcriptional regulation of multiple important pancreatic transcription factors acting both in *cis* and in *trans* and were significantly altered in T2D donor islets. Of particular interest, the downregulation of the *lncRNA* neighbouring the *PDX1* transcription factor gene, *PLUTO*, altered 3D enhancer interactions with *PDX1* and could suggest a shared mechanism of cis-regulator function with *Paupar/Pax6.* Our data reveals complex patterns of transcription factors and binding motifs at novel pancreatic *cis*-regulatory elements. These tune tissue-specific *PAX6* gene expression, can be modulated by common genetic variants and further implicate the *Pax6* locus in pancreas function and diabetes.

## Materials and Methods

### Cell lines

β-TC3 cells were isolated from a mouse insulinoma (27) and were cultured in Dulbecco’s modified Eagle medium (Thermo Fisher Scientific, Waltham, MA) supplemented with 10% fetal calf serum and 1% penicillin–streptomycin at 37°C in 5% CO_2_. Mouse OS25 ES cells were cultured in Glasgow’s minimum essential medium (MEM) (Thermo Fisher) supplemented with 10% fetal calf serum, 1% penicillin–streptomycin, 1% MEM non-essential amino acid solution (Sigma-Aldrich, St. Louis, MO), 1 mm sodium pyruvate (Sigma-Aldrich), recombinant leukaemia inhibitory factor and 0.01 mm 2-mercaptoethanol (Gibco, Thermo Fisher), at 37°C in 5% CO_2_ using standard techniques.

### Chromatin immunoprecipitation

H3K27ac (ab4729, Abcam, Cambridge, UK) or CTCF ChIP (D31H2 XP Rabbit mAb #3418, Cell Signaling, Danvers, MA) was performed as described previously (19) using Protein G Dyna beads (Thermo Fisher Cat# 10003D) with two biological replicates. Primers were designed using Primer3 to PE3 regions with flanking control primers (List of Primers). qPCR was performed using LightCycler® 480 SYBR Green I Master Mix (Cat# 04707516001, Roche, Basel, Switzerland) according to the manufacturer’s guidelines and using a LightCycler® 480 II, with primers at a final concentration of 0.5 μm. Ct values were used to calculate ChIP enrichment at each primer region versus 10% of Input DNA.

H3K27ac ChIPs were validated by qPCR before hybridization to genomic microarrays (720K, NimbleGen, Roche) covering a 66 Mb region around *Pax6* (Chr2: 75 000 000–141 000 000). ChIP and Input samples were amplified (GenomePlex, Sigma-Aldrich), purified (QIAquick, Qiagen) and labelled (NimbleGen Dual-Colour Labelling Kit, Roche Cat. 06370250001). ChIP samples (Cy5) and Input samples (Cy3) were hybridized using a NimbleGen Hybridization and Sample Tracking Control Kit (Roche Cat. 05993776001) according to the manufacturer’s instructions. Slides were washed (NimbleGen Wash Buffer Kit, Roche Cat. 0558450700) and scanned at 2 μm resolution on an MS 200 Microarray Scanner (NimbleGen). Images were processed using NimbleScan (version 2.5); Toedling *et al.* (59) was used for pre-processing, normalization, combining replicates and peak calling of ChIP-chip data. Data was further processed in R by applying a running median (500 bp) and visualized on the UCSC Genome Browser ChIP-Chip tiling array for H3k27ac in β-TC3 cells (GSE116805).

Human PAX4 cDNA (Transgenomics, Omaha, NE) was PCR amplified (Supplementary Material Table S5) and cloned into pcDNA5/FRT/TO/3xFlag with *Hind*III/*Xho*I. pcDNA5/FRT/TO3xFLAG was generated by ligating 3xFLAG [amplified from p3xFLAG-CMV-10 (Sigma-Aldrich); List of Primers] into pcDNA5/FRT/TO at *Afl*II/*Bam*HI. PAX4-FLAG ChIP was performed as described earlier with the following modifications. β-TC3 cells were transfected using Lipofectamine 2000 according to the manufacturer’s protocol (Thermo Fisher) in Opti-MEM, using 12 μg of PAX4-FLAG construct per 10 cm dish, and cells were harvested after 48 h. IPs were performed using mouse monoclonal anti-FLAG M2 (Sigma-Aldrich), mouse IgG as control and sheep anti-mouse IgG M-280 Dynabeads (Thermo Fisher). qPCR was performed on ChIP material with SYBR Select Master Mix (Thermo Fisher Cat# 4472908) on a LightCycler 480 II, standard protocol. To quantify ChIP enrichment, primers were designed to the mouse PE4 element and control regions (List of Primers), and enrichment was calculated as % Input.

### Luciferase reporter assays

β-TC3 cells were grown overnight in a 24-well culture plate. Cells were transfected with luciferase reporter constructs (as described later), human PAX4 or PAX6 cDNA (Transgenomics), and with *Renilla* luciferase pRL-TK (Promega, Madison, WI) as an internal control. Luciferase assays were performed 48 h after transfection using a Dual-Luciferase Reporter Assay System (Promega). Relative luciferase activity was calculated by dividing firefly luciferase signal by *Renilla* luciferase signal and normalizing the resulting value to the relative luciferase activity of the negative control vector. Five biological replicates of each assay were performed. To analyse the expression of PAX4, PAX6- and FLAG-tagged constructs cell extracts were prepared in 4× LDS sample buffer and analysed by protein gel electrophoresis (NuPage, Invitrogen, Thermo Fisher) and western blotting with anti-PAX4 (135598, Abcam), anti-PAX6 (AD1.5.6 and AD2.35) (60), anti-hnRNPU (05-1516, Millipore, Livingston, UK) and anti-FLAG M2 (Sigma-Aldrich) primary antibodies. Luciferase reporter constructs were made by cloning the putative PE4 element (Chr11: 31947517–31948318) into a minimal promoter pGL4.23 vector (Promega). Single-base pair changes corresponding to the SNP variants were introduced into the putative PAX4 motif within the PE4 element by site-directed mutagenesis using a mega-primer approach and confirmed by sequencing.

### Mouse reporter transgenics

Mouse LacZ reporter transgenics were derived as described previously (4). Three founders were obtained for the PE3 element (chr2: 105456479–105456966), two of which showed an expression pattern consistent with previously obtained transiently expressing embryos. Line PE3Z-004 was selected as representative, and multiple embryos from PE3Z-004 male × wild-type female matings were analysed at three developmental stages; E10.5, E11.5 and E17.5. Two stable LacZ lines were established for the PE4 element (chr2: 105390725–105393222; PE4-Z-011 and PE4-Z-029), and representative embryos were analysed at three developmental stages, as previously. All animal experiments were approved by the University of Edinburgh ethical committee (approval ID TR-15-08) and performed under UK Home Office license number PPL 60/3785.

### RNA-seq

Two experimental replicates were generated with two T25 culture flasks of β-TC3 cultured to ∼70% confluency. Total RNA was prepared using the Qiagen RNeasy mini kit (Qiagen) with an on-column DNase I digestion using the Qiagen RNase-Free DNase set as the manufacturer’s guidelines. Ribosomal RNA depletion was performed using the RiboMinus Eukaryote Kit for RNA-Seq (Life Technology, Thermo Fisher) and RiboMinus Concentration module as per manufacturer’s guidelines, with ribosomal depletion was confirmed by gel electrophoresis. Sequencing libraries were prepared using NEBNext mRNA Library Prep Master Mix Set (NEB, Ipswich, MA), following the manufacturer’s protocol, with all size selection performed using Agencourt AMPure XP beads (Beckman Coulter, Brea, CA). Samples were multiplexed suing the NEBNext Multiplex Oligos for Illumina kit to barcode each sample and amplified for 11 cycles. The libraries were analysed using an Agilent high-sensitivity DNA chip bioanalyser (Agilent, Santa Clara, CA). The subsequent samples were processed by the Next Gen Sequencing facility at the Department of Pathology, VU University Medical Centre, Amsterdam, using an Illumina HiSeq 2000 producing single end 50 reads Illumina, San Diego, CA. Reads were aligned to the mm9 genome index using TopHat v2 (68) and processed with Samtools v1.6. Ethical approval was received to re-analyse RNA-seq data from six primary human β-cells from Nica *et al.* (25). For both human and mouse samples, aligned bam files were processed with Subread v1.5 *feature counts* to generate FPKM scores (using total number of aligned of reads and gene length) against hg19 and mm9 RefSeq genes (61). The number of aligned reads scaled by the Bedtools genome coverage tool (62) was used to generate visualization of read distribution across the human and mouse Pax6 loci RNA-seq data β-TC3 (GSE116811).

### Bioinformatics data and analysis

All genomic positions are hg19 or mm9. Primary human histone modification ChIP-seq data was downloaded from the Human Epigenome Atlas (Supplementary Material Table S4). Peak calling was performed using MACS version 1.4.2, with *P*-value cut-off of 0.005 (63). Conserved transcription factor-binding sites within aligned human and mouse sequences were identified with rVista (30). Human–mouse sequence alignment was performed using Clustal Omega. Candidate transcription factor motifs altered by SNPs were identified using HaploReg, and motifs were validated using the Jasper database (64,65). Spearman correlation on neighbouring gene expression was performed in R using RNA-seq reads per kilobase million values from 23 mouse tissue and cell line data sets; 20 samples from (66) and differentiated neurons from (50), and β-TC3 cell RNA-seq were analysed with Geneprof tool (67) (see Supplementary Material Table S4 for details of 23 mouse samples).

### 3C-qPCR

The 3C procedure was based on the methods adapted from (68). A no-ligase control sample was run alongside experimental samples and produced no enrichment in qPCR assays. Adherent β-TC3 and OS25 mESCs were treated with trypsin/Versene and resuspended as a single cell suspension in 10% fetal calf serum in phosphate-buffered saline (FCS/PBS). Cells were counted and 1 × 10^7^ cells were used for the 3C experiment. Cells were fixed in 10% FCS/PBS with 2% (v/v) formaldehyde (Sigma-Aldrich) for 10 min at room temperature. Glycine was added, and cells were centrifuged and lysed in cell lysis buffer [10 mm Tris–HCl, pH 7.5; 10 mm NaCl; 0.2% (v/v) Ipegal, PI]. Cells were centrifuged and resuspended in 500 μl 1.2× NEB 3. 3C samples were incubated with sodium dodecyl sulfate (SDS) at a final concentration of 0.2% for 1 h, before Triton X-100 was added to 2% for 1 h. 1000 U of concentrated *Bgl*II restriction enzyme (NEB Cat# R0144M) was added for overnight digestion at 37°C and 1200 rpm. Digestion efficiency was analysed on 5 μl pre- and post-digested template by gel electrophoresis. The restriction enzyme was deactivated by heating at 65°C for 25 min in 1.6% SDS. Ligation of 3C library was performed in a final volume of 7 ml diluted in 1× T4 DNA ligase reaction buffer (NEB), with 1% Triton X-100. Ligation was performed at 16°C for 4 h at 300 rpm with 3.3 μl of high-concentration DNA ligase (NEB Cat# M0202 M). Cross-links were removed by heating at 65°C overnight with 300 μg of Proteinase K, followed by incubation at 37°C for 1 h with 300 μg RNase A. Samples were purified by two sequential phenol–chloroform extractions (Sigma-Aldrich) and ethanol precipitation and resuspended in 150 μl of 10 mm Tris pH 7.5.

The PE3 and PE4 anchor fragment primers were designed with both the primer and probe to lie within 150 bp of the *Bgl*II cut site. The probes were dual labelled with a 5′ 6-Carboxyfluorescein (6-FAM) fluorophore and a 3′ BHQ1 quencher. The variable fragment primer panel (primer list) was designed to lie within 100–150 bp of the target *Bgl*II restriction site using an *in silico* digest of the mouse genome across the *Pax6* regions (mm9 build). The panel of variable fragment primers was validated on a random ligation template (RLT; design below) using the anchor primers and constant probe primer to confirm each primer and probe efficiency across a range of DNA concentrations.

Bacterial artificial chromosome (BAC) DNA for the Pax6 locus (RP23-281P3, Chr2: 105 427 870–105 581 806) and P1-derived artificial chromosome (PAC) for the control *Ercc3* region (PAC 334G18) were used to generate an RLT for the region of interest, to produce values of the standard curves for each primer combination. BAC and PAC DNA was prepared using a standard alkaline lysis mini-prep method. The RLT was prepared (69), with equimolar amounts of BAC and PAC DNA digested with *Bgl*II as for the 3C protocol, phenol–chloroform extracted and precipitated. The DNA was ligated at high DNA concentrations to promote intramolecular ligation events and purified. Standard curves were generated using the RLT on the primer panel as described by (69), with serial 5-fold dilutions of RLT used to produce standard curves for all variable primers with the probe and constant fragment primer. Digested and ligated genomic DNA was used to keep the sample DNA concentration in the qPCR reaction constant, and the probe qPCR protocol was performed as 3C samples qPCR. Absolute quantification was performed using a LightCycler 480 II (Roche) using LightCycler 480 software to generate the standard curve efficiency, slope and intercept values. Four qPCR reactions were performed for each primer sample combination, using QuantiTect Probe PCR master mix standard protocol (Qiagen Cat# 204341), the probe at a final concentration of 0.15 μm and primers at 0.5 μm and the LightCycler 480 program described by Hagège *et al.* (68), detecting 6-FAM signal. Two independent biological experiments were performed, each generating individual Ct values, and calculations were performed as in (68) with each Ct value used to calculate a relative cross-linking frequency using the parameters of the standard curve and then normalized to *Ercc3* background interactions to allow comparison between samples. The final relative cross-linking and SEM calculation was performed on the mean relative cross-linking across both experimental replicates, and this was plotted as a function of the distance from the anchor probe to the test primer.

### TALEs

TALEs were assembled using a modular assembly system) (41) in which specific RVD DNA-binding domain is constructed through assembly of three 4-mer modules and one 3-mer plasmid module, linearized with *Bsm*BI and cloned into pTAL-VP64-eGFP (42). The PE3 and PE4 regulatory element target sequence were identified using the TALE nucleotide targeter 2.0 tool (70) and were targeted to a 16 bp sequence in the core element sequence (PE4_TAL: TCCTCAGGCCATGCAT, chr2: 105392464–105392479; PE3_TAL: TCGAGCTAATCCTCTT, chr2: 105456674–105456689). To generate SID4X TALEs, the VP64 sequence was removed using (*Bam*H1/*Nhe*I digestion) and replaced with the SID4X sequence (see primers).

Mouse ES and β-TC3 cells were seeded at ∼70–80% confluency in 60 mm cell culture dishes 24 h before transfection and transfected in Opti-MEM with Lipofectamine 3000 (Thermo Fisher) using a standard protocol with 5 μg of plasmid and incubated for 48 h (β-TC3) and 36 h (mESC). Cells were washed in PBS, trypsinized and flow sorted for eGFP expression, and GFP-positive cells were collected. Flow sorting was performed on a BD FACSAria2 SORP cell sorter by the MRC HGU FACS facility; GFP data was collected using a 488 nm excitation laser and a 525/50 nm bandpass emission filter, and BDFACS Diva software version 6.1.3 was used for data collection. GFP-positive cells were collected and RNA extracted using a RNeasy mini Kit (Qiagen), with GFP-negative mock-transfected cells as control; all RNA samples were digested with DNase I on column (Qiagen). RNA concentration was measured and corrected for all samples and reverse transcribed to cDNA using SuperScript III (Thermo Fisher) using a standard first-strand synthesis protocol with Oligo(dT) primers (Promega), for two biological replicates. Real-time qPCR was used to measure transcript levels using a LightCycler 480 II (Roche), with SYBR Select Master Mix (Thermo Fisher) following the manufacturer’s protocol with primers at 0.5 μm. Gene-specific primers were designed to Ensemble cDNA transcripts (List of Primers); we were unable to design primers which could assay Pax6OS1. The log2-fold change was calculated using the ΔΔCT method against mock-transfected controls, with 18S as a housekeeping gene.

#### List of Primers

##### Pax4 ChIP qPCR

ChrX_Con: TTCTGGGGTTTGTGCATGTG(f) AGAGTAGAAGGACGGTATTGGT(r)
PE4_1: TAGCCGGTGTTCCATTGTCT(f) GCTAGTGTTTAAACCGCTCCA(r)
PE4_2: GCCAACCAGACAATCTTCAGT(f) GCTGGGCTGTAATTTGCTGA(r)
PE4_3: GGAGCGGTTTAAACACTAGCA(f) GAGCTTCTCTGGCAGCCTT(r)
PE4_Con: ATGTGCAGCTATCCCCATGT(f) TGTGGAATGCTCAGCCCTAA(r)
Chr2_Con: GTGGCACATCACAAATGCTC(f) TCTCCAGTCTAACACTTGGCAAT(r)

##### CTCF ChIP qPCR

Hum.PE4.1: CGCTAGTTTCAATTTGGCTGT(f) TGAATAGCGGCAAAGATCCTG(r)
Hum.PE4.2: TGAAGATACCTGGATGAAGCACT(f) TGTGAATGAATAGCGGCAAAGA(r)
Luc.IP.1: TTCGACCGGGACAAAACCAT(f) ATCTGGTTGCCGAAGATGGG(r)
Luc.IP.2: TTCGGCAACCAGATCATCCC(f) GTACATGAGCACGACCCGAA(r)
PE3_1: CCAGTGCTCTGGGCTACAAT(f) AAGACGCCAGGAAGAGGATT(r)
PE3_2: AATCCTCTTCCTGGCGTCTT(f) GGAAGGCTCTGTCCCTCTTT(r)
Con_+1kb: TGTTTTGGGGTCTCCTGAAG(f) CATGGACTAACAATGCTTCTCCT(r)
Con_-1kb: AAGAGGACTCAGCGAAACCA(f) TGACCTCATGCCAACTCATC(r)

##### Taqman probes

PE3_probe: CCTCTTCTTTCACAGTGTTCCCTGA
PE4_probe: CCTGCCTTGAAAACTCTTCTCGTCTCTG
Ercc3_probe : CCAGACCAGAGAGCGGAGACC

##### 3C primers

3C_PE4.F: GGTGAGTCTTTACATGTGGGG
3C_1.F: TCCCACCATCCAAATTCTGT
3C_2.F: GCCATCTCTCTAGCCCCTTT
3C_3.F: CCTCTTCTGTCCAGGCTTTG
3C_PE3.F: AGCAAATGTGTGACCGTGAG
3C_4.F: ATCCACCCACCTCCTTATCC
3C_5.F: CTCTTTCTGGTTTGCGGTAT
3C_6.F: CTGTTCTTCCTCTGAAACCTG
3C_7.F: ACAGTGGCACGTTGGATATG
3C_8.F: TGTGTGCAAATGAAGGCTCT
3C_9.F: TGACCTGCAAGAAGACACAGA
3C_10.F: CGCTTTGATTCTAGCCAGAC
3C_11.F: GTGTAATTGAGGGAAATGGAGTTGAA
3C_12.F: GGCCAGTTTGACACACCTTT
3C_13.F: CCCCAACCTTTGTACTCAGC
3C_14.F: TCTTTTGCCCAGAGATGAGC
3C_15.F: GGAAAGGCACTTGGAAATGA
3C_16.F: CTGGTGACCATCCACTCTCC
3C_17.F: TGAGAGGACCCATTATCCAGA
3C_errc3_1.F : GGCTGAGAGTGATGCTGCTA
3C_errc3_2.F: CGGTAAATCTCCTCCCAAAT

##### Cloning

hPAX4: TCGCAAGCTTATGAACCAGCTTGGGG(f) TGCCCTCGAGTTATTCCAAGCCATACAG(r)
3xFLAG: ACGACTTAAGGGCGCGCCACCATGGACTACAAAGACC(f) GTCAGGATCCTCTAGAGTCGAC(r)
SID4X: ACGAGGATCCGGCTCCGGGATGAACATCCAG(f) GTCAGCTAGCTCTACTGGGCAGCATAGAGG(r)

##### TAL RT-qPCR

Pax6 ex6-7: AGTTCTTCGCAACCTGGCTA(f) GTGTTCTCTCCCCCTCCTTC(r)
Pax6 ex4-5: CGTGCGACATTTCCCGAATT(f) CTTGGCTTACTCCCTCCGAT(r)
Paupar: TGCTCTTCTGTCTAGGGTGC(f) AACTTCATCCAAAAGGCCGG(r)
Oct4: CGAGAACAATGAGAACCTTC(f) CCTTCTCTAGCCCAAGCTGAT(r)
18s: GTAACCCGTTGAACCCCATT(f) CCATCCAATCGGTAGTAGCG(r)
Immpl1.ex3_4: GCTTTTCGACTTGCTGGCTA(f) TGTCGGCTAAGATTTTCTGCA(r)
Rcn1.ex2_3: AGAATACAAGCAGGCCACCT(f) GCAGAAAGGCAGTGAACTCC(r)
Elp4.ex3_4: CATGGCAGAAGGAATCATCA(f) GCTCTGGGGTTTTAGCACTG(r)

## Supplementary Material

Supplementary Data
