# Supplementary material for: Functional characteristics of novel pancreatic Pax6 regulatory elements
Source: Hum Mol Genet. 2018 Aug 10;27(19):3434–48. doi: 10.1093/hmg/ddy255 (PMC6140780; doi:10.1093/hmg/ddy255)
Supplement: Supplementary Data [file ddy255_supp.pdf]

Supplementary Information Legends

Supplementary Figure 1. **Human PE3 and PE4 regulatory element tissue specificity.** A. UCSC browser image of 650 kb human PAX6 locus with coloured density plots of H3K4me1 ChIP-seq data for 48 human tissue types from Human Epigenome Atlas data. For details of tissue legend see Table S4.

Supplementary Figure 2. PE3 and PE4 LacZ reporter expression pattern in the embryonic brain. A. Dissected E17.5 PE3 brain with staining in olfactory bulb (ob), neocortex surface (nco), midbrain tectum (mt), pontine hindbrain (poh), and spinal cord (sc). B. Coronal section displaying E17.5 PE3 staining rostral to caudle, ob, the alar plate region of telencephalon (tc), alar plate of thalamus pre-thalamus region (ptr), mt, cerebellum (cb), hindbrain (hb) and sc. C. Ventral view of dissected PE4 E17.5 brain, showing nerve tracts in the forebrain corresponding to the lateral olfactory tracts (lot) and staining in the cerebellar region and pontine grey nuclei (pgn) of the hind brain.

Supplementary Figure 3. Sequence conservation and TF motif of PE3 and PE4 regulatory elements. A. Sequence alignment between human and mouse PE3 enhancer showed 58.33% identity (yellow), SNPs marked in red. CTCF motif (orange) identified using CTCF binding Site Database prediction tool (1) and position of PE3 TAL effector targeting site. B. ChIP assay for CTCF in β-TC3 cells, evaluated by qPCR, using primer sets to the mouse PE3 element and control +/- 1 kb from PE3. Values are displayed as percentage of input sample, with two independent experimental replicates. Error bars +/- SEM. C. PE4 enhancer showed 74.2% identity (yellow) between human and mouse with SNPs marked in red. Pax4 motif marked in orange and PE4 TAL effector targeting site.

Supplementary Figure 4. PE3 and PE4 heterozygosity analysis. Plot of average heterozygosity calculated using dbSNP data base (version 141). Yellow areas mark position of PE3 and PE4 regulatory elements.

Supplementary Figure 5. PAX4 PE4 motif region showing multiple species sequence conservation. Human PE4 DNA sequence (53 bp) aligned to 59 vertebrate species. Conservation data from UCSC Vertebrate Multiz Alignment 100 Species. Putative regulatory SNP marked in red/yellow.

Supplementary Figure 6: Gene expression correlation and HiC map over *Pax6* locus. A. Spearmen correlation co-efficient heatmap of RNA-Seq RPKM values for 23 mouse cell lines and tissues over *Pax6* genomic region, see Table S4 and methods for details. B. HiC heatmap from mESC over 1.7 Mb *Pax6* region, with TADS and gene location marked. HiC data from (2) visualized with the HiC browser (3).

Table S1: Gene expression profiles in six human beta cell transcriptomes and two replicates of β-TC3 at the Pax6 locus. Summary FPKM values of 6 purified primary human beta cell and two mouse β-TC3 replicate RNA-seq experiment, human and mouse gene names listed. FPKM calculated with subread feature counts aligned to UCSC RefSeq Genes. Human transcriptomes from [30] were used with ethical permission from European Bioinformatics Institute, accession number EGAS00001000442.

Table S2. Conserved transcription factor binding sites within the PE3 element. Conserved TFs were identified between the two aligned human and mouse PE3 sequences with rVista tool [34]. Pancreas FPKM (Fragments Per Kilobase of transcript per Million mapped reads) represents RNA-seq reads from adult pancreas gene expression data collated from the Human protein atlas [35].

Table S3. Conserved transcription factor binding sites within the PE4 element. Conserved TFs were identified between the two aligned human and mouse PE4 sequences with rVista tool [34]. Pancreas FPKM (Fragments Per Kilobase of transcript per Million mapped reads) represents RNA-seq reads from adult pancreas gene expression data collated from the Human protein atlas [35].

Table S4. Details of ChIP-seq, ChIP-chip and RNA-seq datasets used in study. Primary human histone modification ChIP-seq data sets from the Human Epigenome Atlas consisting of H3K4me1 data for 48 distinct cell types, three H3K27ac data sets for pancreatic Islet, whole pancreas and small intestine, H3K27ac and RNA-seq datasets β-TC3 cells (generated for this study), and primary human β-cell RNA-seq [30], RNA-seq from 21 mouse tissue and cell line from (4) and (5).

Tables

Table S1: Gene expression profiles in six human beta cell transcriptomes and two replicates of β-TC3 over the *PAX6/Pax6* locus.

Gene	*PAX6/Pax6*	*PAUPAR/Paupar*	*PAX6-AS1/Pax6Os1*	*RCN1/Rcn1*
h-βcell 1	19.6	5.2	9.1	79.5
h-βcell 2	25.8	3.4	19.5	70.1
h-βcell 3	23.0	6.4	7.5	78.9
h-βcell 4	23.5	6.2	9.4	61.1
h-βcell 5	18.8	6.4	6.9	94.5
h-βcell 6	24.7	6.5	9.0	65.0
m β-TC3 R1	110.6	2.2	6.2	15.8
m β-TC3 R2	109.1	2.2	6.1	16.8

Table S2. Conserved PE3 element transcription factor binding sites.

Gene	TF DNA motif	TF Motif Name	Pancreas expression (FPKM)
EGR2	cccaCTCCCAC	EGR_Q6	1.6
GTF2B	cctCATTg	GEN_INI_B	9.8
SOX9	cctcATTGTTttcc	SOX9_B1	18.05
Sox family (SOX13, SOX11, SOX2, SOX15, SOX7, SOX8, SOX14,SOX13, SOX17)	cTCATTGTTttcc	SOX_Q6	6.4, 0, 0.05, 0.2,1.5, 0.5, 0, 6.6, 0.15
POU5F1	taTTCAAATGCAAAc	OCT4_01	0.8
DDIT3	aaaTGCAAAcacc	CHOP_01	11.1
FOXA1	aaatgcAAACA	HNF3ALPHA_Q6	0.35

POU2F1	aATGCAAACa / cCTTTGCATt	OCT1_B	1.2
TFCP2	GCAAACACCAG	CP2_01	7.3
ELF2	cacCAGGAGGcgcctttg	NERF_Q2	10.35
ZBTB14	aggaggCGCCTtt	ZF5_B	5.9

Table S3. Conserved PE4 element transcription factor binding sites.

Gene name	Motif Sequence	TF	Pancreas expression (FPKM)
IKZF2	ctatGGGAtaa	HELIOSA_01	0.8
RBPJ	tGTGGGAA	RBPJK_Q4	12.55
STAT1	gggAAatt	STAT1_03	22.45
HMGA1	GGAAAtt	HMGIY_Q6	11.1
HOXA4	gaAATTAG	HOXA4_Q2	0.21
RORA	gaAATTAGGTTAt	RORA2_01	6.85
HOXA3	gaaattAgg	HOXA3_01	0.15
POU2F1	attagGTTATGCAAATACTttgc /tagGTTATGCAAATACttt/ tATGCAAATa	OCT1_04/V$OCT1_01/OCT1_B	1.2
CEBPD	aggttatGCAAa	CEBP_Q3/CEBP_Q2_01/CEBPDELTA_Q6	5.95
HLF	gTTATGCAAa	HLF_01	1.65
POU3F2	ATAAACATATGCAT	POU3F2_01/V$POU3F2_02	0
AIRE	tattatCGATTTATACCtatttcttt	AIRE_01	0
BHLHE40	cttTCATGTGCtct	STRA13_01	37
TCF11:MafG hetrodimer	cttaagTGCCTCAGCAaattac	TCF11MAFG_01	27.5/4.2
NKX2-2	tTAAGTGCCT	NKX22_01	2.1
NKX2-5	TTAAGTG	NKX25_01	0
MYOD	tgcCTCAGCAAATTACAGCCCAGCCAgcc	MYOGNF1_01	0
BACH1	gcaATGAGGCATtcc	BACH1_01	6.65
TEAD1	CATTCC	TEF1_Q6	8.85
FOXC1	caactGTAAATAaaca	FREAC2_01	1.95
FOXJ2	ctgtaaATAAACAtatgc	FOXJ2_01	3.85
FOXI1/FOXJ1/FOXF1	tAAATAAACAtat	HFH3_01	0.1/0.45/0.65

TAL1	AGCAGAAGGa	TAL1_Q6	0.1
IRF1	ggGTTTGGTTTtc	IRF1_01	1.2
HSF1	tTTCTAGAGATGGCttc	HSF1_Q6	8.2
MZF1	**G/C**TATGGGAAA	MZF1_1-4	5.5
KAISO	ATA**G/C**CAGGATCTTTG	ZBTB33	4.2
Pax4	AGATATTTGCATAACCTAATTTCCC ATA**G/C**C	Pax4	0.0

Table S4. Details of ChIP-seq, ChIP-chip and RNA-seq datasets used in study.

Name	Sample	Accession	Cell type / Tissue	Species and origin
AG	ChIP-seq H3K4me1	GSM1059447	Adrenal Gland	Human primary
AK	ChIP-seq H3K4me1	GSM773001	Adult Kidney	Human primary
AL	ChIP-seq H3K4me1	GSM621654	Adult Liver	Human primary
Aorta	ChIP-seq H3K4me1	GSM1013150	Aorta	Human primary
Bladder	ChIP-seq H3K4me1	GSM1059450	Bladder	Human primary
BAG	ChIP-seq H3K4me1	GSM772962	Brain Angular Gyrus	Human primary
BAC	ChIP-seq H3K4me1	GSM772830	Brain Anterior Caudate	Human primary
BCG	ChIP-seq H3K4me1	GSM670033	Brain Cingulate Gyrus	Human primary
BHM	ChIP-seq H3K4me1	GSM916039	Brain Hippocampus Middle	Human primary
BITL	ChIP-seq H3K4me1	GSM772992	Brain Inferior Temporal Lobe	Human primary
BMFL	ChIP-seq H3K4me1	GSM773014	Brain Mid Frontal Lobe	Human primary
BSN	ChIP-seq H3K4me1	GSM669941	Brain Substantia Nigra	Human primary
BF	ChIP-seq H3K4me1	GSM1127065	Breast Fibroblast Primary Cells	Human primary
CD14	ChIP-seq H3K4me1	GSM1102793	CD14 Primary Cells	Human primary
CD15	ChIP-seq H3K4me1	GSM773038	CD15 Primary Cells	Human primary
CD19	ChIP-seq H3K4me1	GSM1027296	CD19 Primary Cells	Human primary
CfBMDMSC	ChIP-seq H3K4me1	GSM670024	Chondrocytes from Bone Marrow Derived Mesenchymal Stem Cells	Human primary
CM	ChIP-seq H3K4me1	GSM621670	Colonic Mucosa	Human primary
CSM	ChIP-seq H3K4me1	GSM772972	Colon Smooth Muscle	Human primary
DM	ChIP-seq H3K4me1	GSM916017	Duodenum Mucosa	Human primary
Esophagus	ChIP-seq H3K4me1	GSM1120349	Esophagus	Human primary
FAG	ChIP-seq H3K4me1	GSM1102796	Fetal Adrenal Gland	Human primary
FB	ChIP-seq H3K4me1	GSM706850	Fetal Brain	Human primary
FH	ChIP-seq H3K4me1	GSM772732	Fetal Heart	Human primary
FIL	ChIP-seq H3K4me1	GSM1058775	Fetal Intestine Large	Human primary
FIS	ChIP-seq H3K4me1	GSM1058776	Fetal Intestine Small	Human primary
FL	ChIP-seq H3K4me1	GSM621429	Fetal Lung	Human primary
FML	ChIP-seq H3K4me1	GSM1058777	Fetal Muscle Leg	Human primary

FP	ChIP-seq H3K4me1	GSM1102795	Fetal Placenta	Human primary
FS	ChIP-seq H3K4me1	GSM1102794	Fetal Stomach	Human primary
FT	ChIP-seq H3K4me1	GSM1027298	Fetal Thymus	Human primary
Gastric	ChIP-seq H3K4me1	GSM1013147	Gastric	Human primary
LV	ChIP-seq H3K4me1	GSM906404	Left Ventricle	Human primary
Lung	ChIP-seq H3K4me1	GSM1059443	Lung	Human primary
MS	ChIP-seq H3K4me1	GSM621752	Muscle Satellite Cultured Cells	Human primary
Ovary	ChIP-seq H3K4me1	GSM1013148	Ovary	Human primary
Pancreas	ChIP-seq H3K4me1	GSM1013149	Pancreas	Human primary
Pancreas	ChIP-seq H3K4me1	GSM910576	Pancreas	Human primary
PI	ChIP-seq H3K4me1	GSM1127087	Pancreatic Islets	Human primary
PI	ChIP-seq H3K4me1	GSM537642	Pancreatic Islets	Human primary
PFF	ChIP-seq H3K4me1	GSM941717	Penis Foreskin Fibroblast Primary Cells	Human primary
PFK	ChIP-seq H3K4me1	GSM941736	Penis Foreskin Keratinocyte Primary Cells	Human primary
RSM	ChIP-seq H3K4me1	GSM669964	Rectal Smooth Muscle	Human primary
RA	ChIP-seq H3K4me1	GSM915335	Right Atrium	Human primary
RV	ChIP-seq H3K4me1	GSM1059445	Right Ventricle	Human primary
SC	ChIP-seq H3K4me1	GSM956020	Sigmoid Colon	Human primary
SI	ChIP-seq H3K4me1	GSM956019	Small Intestine	Human primary
Spl	ChIP-seq H3K4me1	GSM1120351	Spleen	Human primary
StM	ChIP-seq H3K4me1	GSM621642	Stomach Smooth Muscle	Human primary
Thymus	ChIP-seq H3K4me1	GSM1059446	Thymus	Human primary
Pan Islet H3K27ac	ChIP-seq H3K27ac	GSM1127061	Pancreas Islet	Human primary
Small In H3K27ac	ChIP-seq H3K27ac	GSM915330	Small Intestine	Human primary
β-TC3 H3K27ac	ChIP-Chip H3K27ac	GSE116805	Pancreatic Beta cell line	Mouse cell line
β-TC3 RNA-seq	RNA-seq	GSE116811	Pancreatic Beta cell line	Mouse cell line
h-βcell	RNA-seq	EGAS00001000442	Six Sorted primary human Beta-cells	Human primary
Neurons	RNA-seq	SRP009040	ES-cell Differentiated	
Bone Marrow	RNA-seq	SRP006787		Mouse cell line
Brain	RNA-seq	SRP006787	Whole brain	Mouse tissue

Cerebellum	RNA-seq	SRP006787		Mouse tissue
Cortex	RNA-seq	SRP006787		Mouse tissue
Heart	RNA-seq	SRP006787		Mouse tissue
Kidney	RNA-seq	SRP006787		Mouse tissue
Liver	RNA-seq	SRP006787		Mouse tissue
Lung	RNA-seq	SRP006787		Mouse tissue
Spleen	RNA-seq	SRP006787		Mouse tissue
MEF	RNA-seq	SRP006787	Mouse embryonic fibroblasts	Mouse tissue
mESC	RNA-seq	SRP006787		Mouse cell line
Brain embryo	RNA-seq	SRP006787	E14.5 embryo	Mouse cell line
Heart embryo	RNA-seq	SRP006787	E14.5 embryo	Mouse tissue
Limb embryo	RNA-seq	SRP006787	E14.5 embryo	Mouse tissue
Liver embryo	RNA-seq	SRP006787	E14.5 embryo	Mouse tissue
Intestine	RNA-seq	SRP006787		Mouse tissue
Olfactory	RNA-seq	SRP006787		Mouse tissue
Placenta	RNA-seq	SRP006787		Mouse tissue
Thymus	RNA-seq	SRP006787		Mouse tissue
Testes	RNA-seq	SRP006787		Mouse tissue
Muscle	RNA-seq	SRP006787		Mouse tissue

Supplementary References

1. Ziebarth,J.D., Bhattacharya,A. and Cui,Y. (2012) CTCFBSDB 2.0: a database for CTCF-binding sites and genome organization. *Nucleic Acids Res.*, **41**, D188–D194.

2. Dixon,J., Selvaraj,S., Yue,F., Kim,A., Li,Y., Shen,Y., Hu,M., Liu,J.S. and Ren,B. (2012) Topological domains in mammalian genomes identified by analysis of chromatin interactions. *Nature*, **485**, 376–380.

3. Wang,Y., Zhang,B., Zhang,L., An,L., Xu,J., Li,D., Choudhary,M.N., Li,Y., Hu,M., Hardison,R., *et al.* (2017) The 3D Genome Browser: a web-based browser for visualizing 3D genome organization and long-range chromatin interactions. *bioRxiv*, 10.1101/112268.

4. Shen,Y., Yue,F., McCleary,D.F., Ye,Z., Edsall,L., Kuan,S., Wagner,U., Dixon,J., Lee,L., Lobanenkov,V.V., *et al.* (2012) A map of the cis-regulatory sequences in the mouse genome. *Nature*, **488**, 116–120.

5. Bergsland,M., Ramsköld,D., Zaouter,C., Klum,S., Sandberg,R. and Muhr,J. (2011) Sequentially acting Sox transcription factors in neural lineage development. *Genes Dev.*, **25**, 2453–2464.

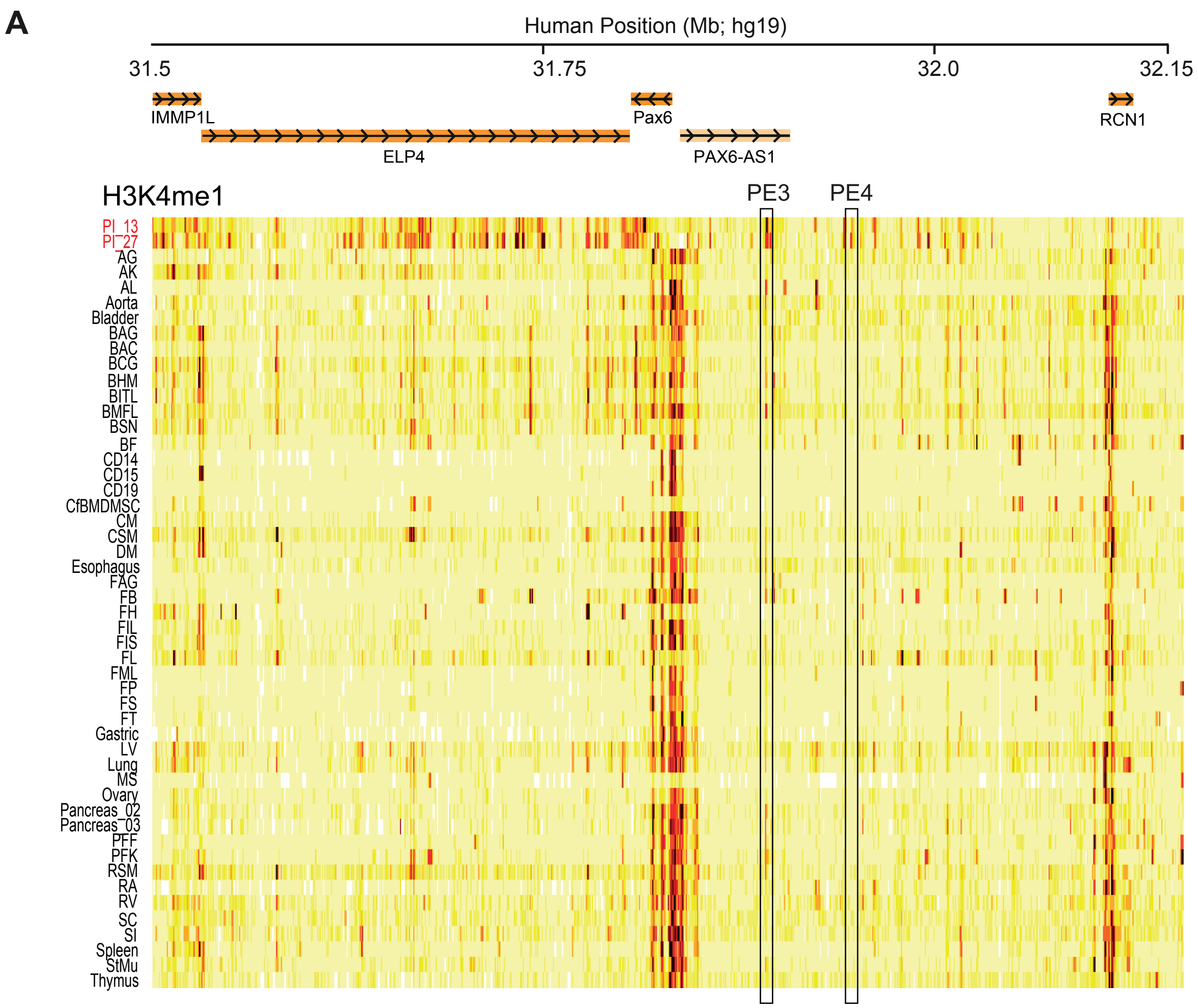

Supplementary Figure 1

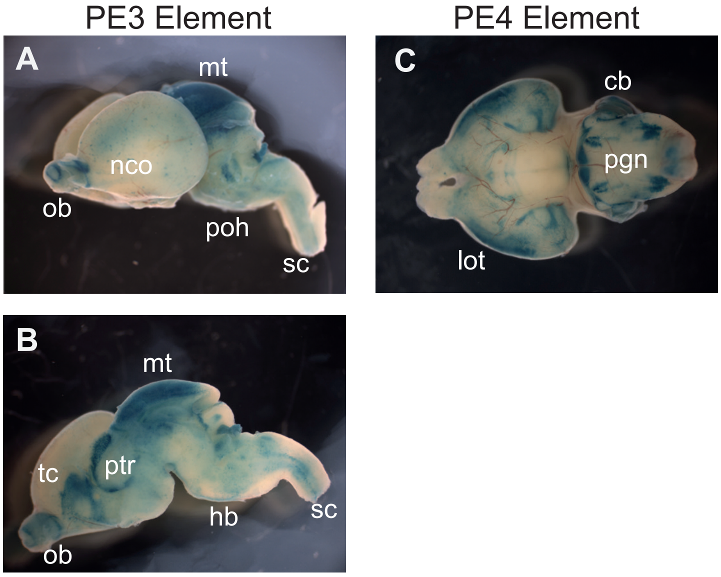

Supplementary Figure 2

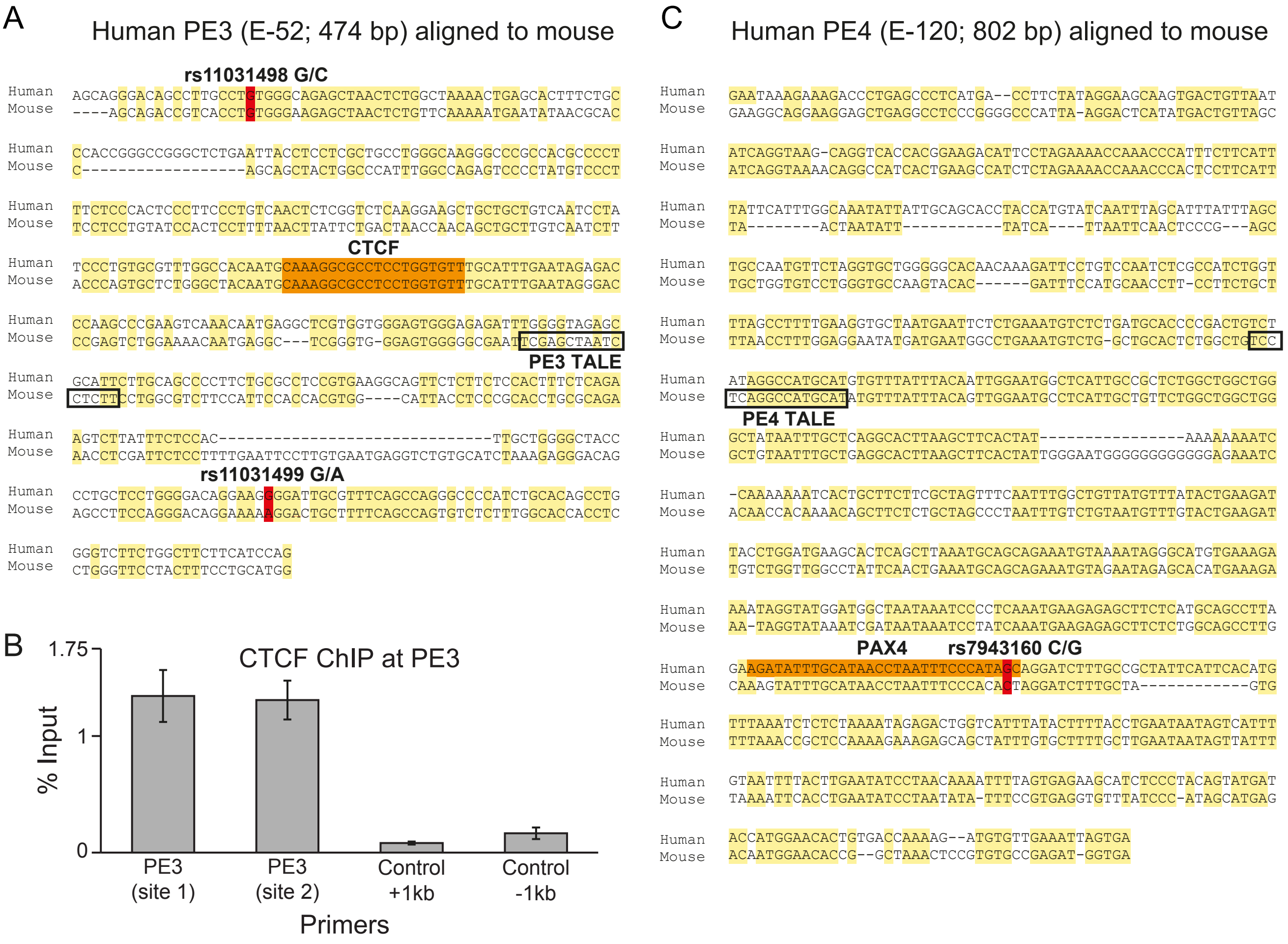

Supplementary Figure 3

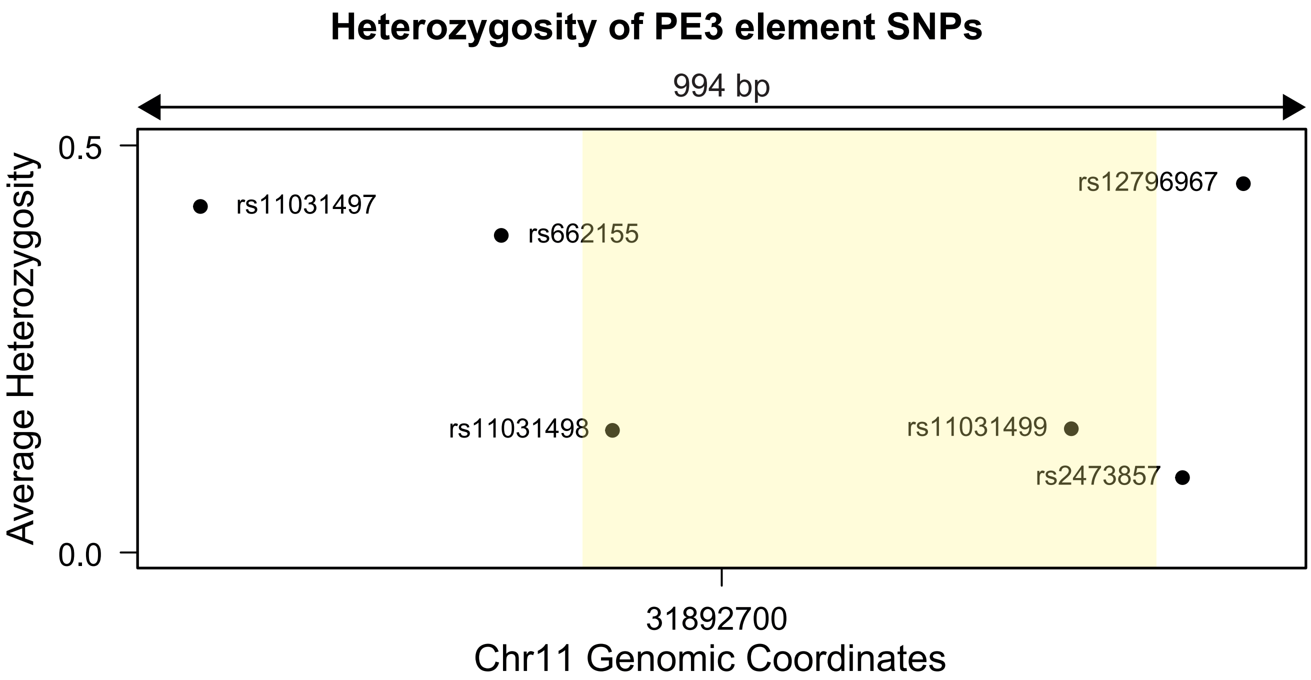

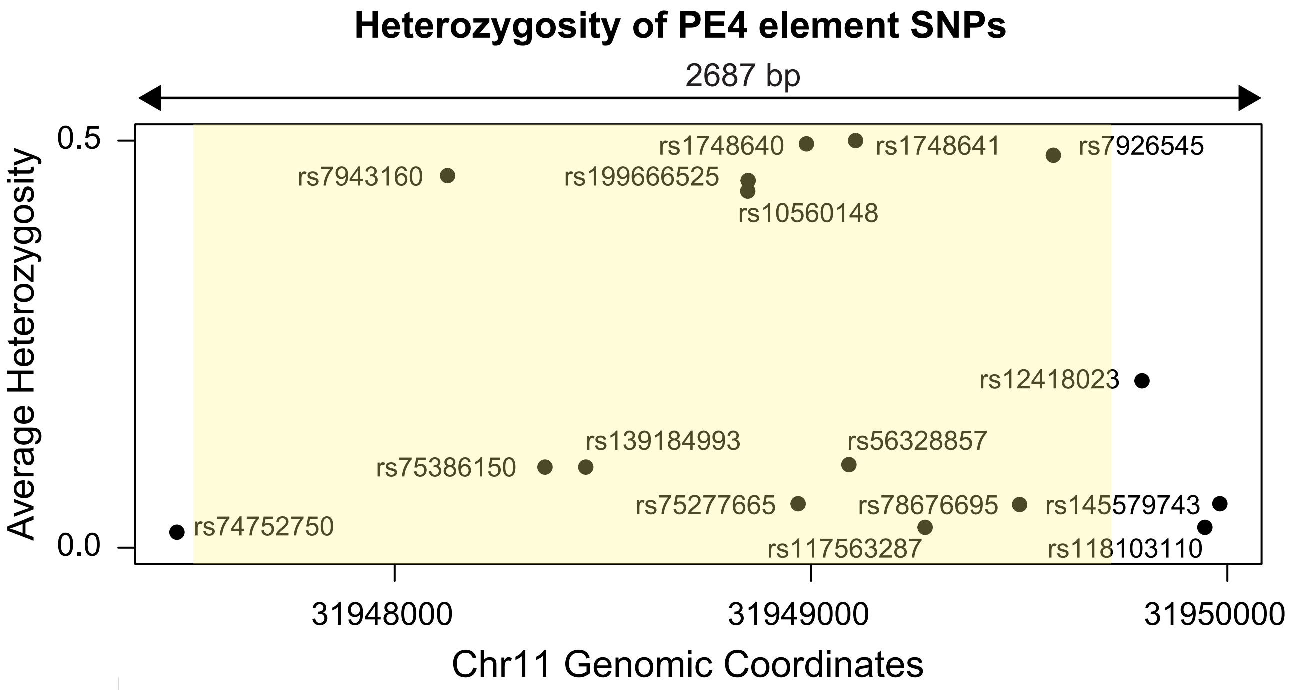

Supplementary Figure 4

Pax4 Motif

```
Human                 aagatatttgcataaccta---attt-ccca-ta-gcag--gatctttg----ccgct-----attcatt
Chimp                 ...............g...................c..................................
Gorilla               ...................................c..................................
Orangutan             ...g...........g...................c..................t...............
Gibbon                ...g...............................c..................................
Rhesus                ...g..............c.........aa.....c..a................a..............
Crab-eating macaque   ...g..............c.........aa.....c..a................a..............
Baboon                ...g..............c.........aa.....c..a................a..............
Green monkey          ...g..............c.........aa.....c..a................a..............
Marmoset              ...g...............................c..................t..c...........c
Squirrel monkey       .............................t...t.ct....................c.......c....
Bushbaby              ..ac............................c..c......g...........t.t.............
Chinese tree shrew    ...gc........-....g..........tg..c.c.--.......cca.....t.tc.......c..c.
Squirrel              ...g.........................tg.c..c..a..t..tc........t..........c....
Egyptian jerboa       ................................c..c..................t.t........c....
Prairie vole          ..ag......................c.....c..c.g.........ca.....t.--.....-------
Chinese hamster       ..ag............................c..c.g................t.--.....-------
Golden hamster        ..ag............................c..c.g................t.--.....-------
Mouse                 ..ag............................c..ct.................ta--.....-------
Rat                   ..ag............................c..c..................t.--.....-------
Naked mole-rat        ...gcg..c..c.c...gg......c...tg.c..c......g...........t........c.ctg..
Guinea pig            ct.g............................cg........c...gca.....a........g.c....
Chinchilla            ...c.g.........t..c.............cc.c......g...c.......t........-------
Brush-tailed rat      c..g.g..c...cg..................cc.c.--.......g......tt..........ctgc.
Rabbit                .................cg.......c.....c..ct..........ca....tt.t.......ag....
Pika                  .................c........ct....c..at....a.....ca....gt..........a....
Pig                   ...g............................c..ca.................t..........c.c.-
Alpaca                ..ag.......g.gtg.......-c.......c..c.g................tct........c.t..
Bactrian camel        ...g.......g.gtg.......-c.......c..ctg................tct........ctg..
Dolphin               ...g............................c..c..................t.t........c....
Killer whale          ...g............................c..c..................t.t........c....
Tibetan antelope      ...g...........a..g...........c.c.tc.....a....c.......tct........c....
Cow                   ...g...........a..g...........c.c.tc.....a....c.......tct........c....
Sheep                 ...g...........a..g........t..c.c.tc.....a....c.......tct........c....
Domestic goat         ...g...........a..g...........c.c.tc.....a....c........ct........c....
Horse                 ...g..........c.........c...t.c..g.c......g..g.........at......c.c....
White rhinoceros      ...g............a.......c...-...cg.c.......c.........tg........c.c..c.
Cat                   ...g.........................tg.ct.c.g................t.t........c....
Dog                   ...g.........................t...t.c..................a.t........c...g
Ferret                ...g.........................tg.ct.ctgc..a....c.........t.......actg.g
Panda                 c..g.........................tg.ct.c..c...............t.t........c...g
Pacific walrus        ...g.........................tg.ct.c..cga......c......t.t........c...g
Weddell seal          ...g.........................tg.ct.c..cga......c......t.t.......cc...g
Black flying-fox      ...g............................c..c..a....---........t.t........c....
Megabat               ...g............................c..c..a....---........t.t........c....
David's myotis bat    ...g............................c..c.....a.---........t.t.............
Microbat              ...g............................c..c.....a.---........t.t.............
Big brown bat         ...g............................c..c.....a.---........t.t........c....
Hedgehog              g..g...c...................t...g.g.c........t.a.......tat.gaaac..ct..c
Shrew                 .............................t..--.----..--------....--at.......gc..c.
Star-nosed mole       ..tg...........................cc..-........a.........t.t........c..c.
Elephant              t..g..c......t...............tg.cc.c..t.....t...t....tt.t.............
Cape elephant shrew   tg.g.........t.............g....cctctct..tt.t...t.....tat.............
Manatee               t..g.........t..................ct.c..t.....t...t.....t.t.............
Cape golden mole      t..g.........c..................cc.c..t.....t...ttccc.t.t.............
Tenrec                t..g.........tg.................cc.c.gt.....t...t.....t.t.............
Aardvark              t..g.........t..................cc.t..t..a..t...tt....t.t.............
Armadillo             g..g........g..........-.....tg.c..c.................tt.t.......cc...g
Platypus              ....a....t...gtg...ttt...c.tttt.ct.ct.t..t..t...a.....atc......-------
```

Supplementary Figure 5

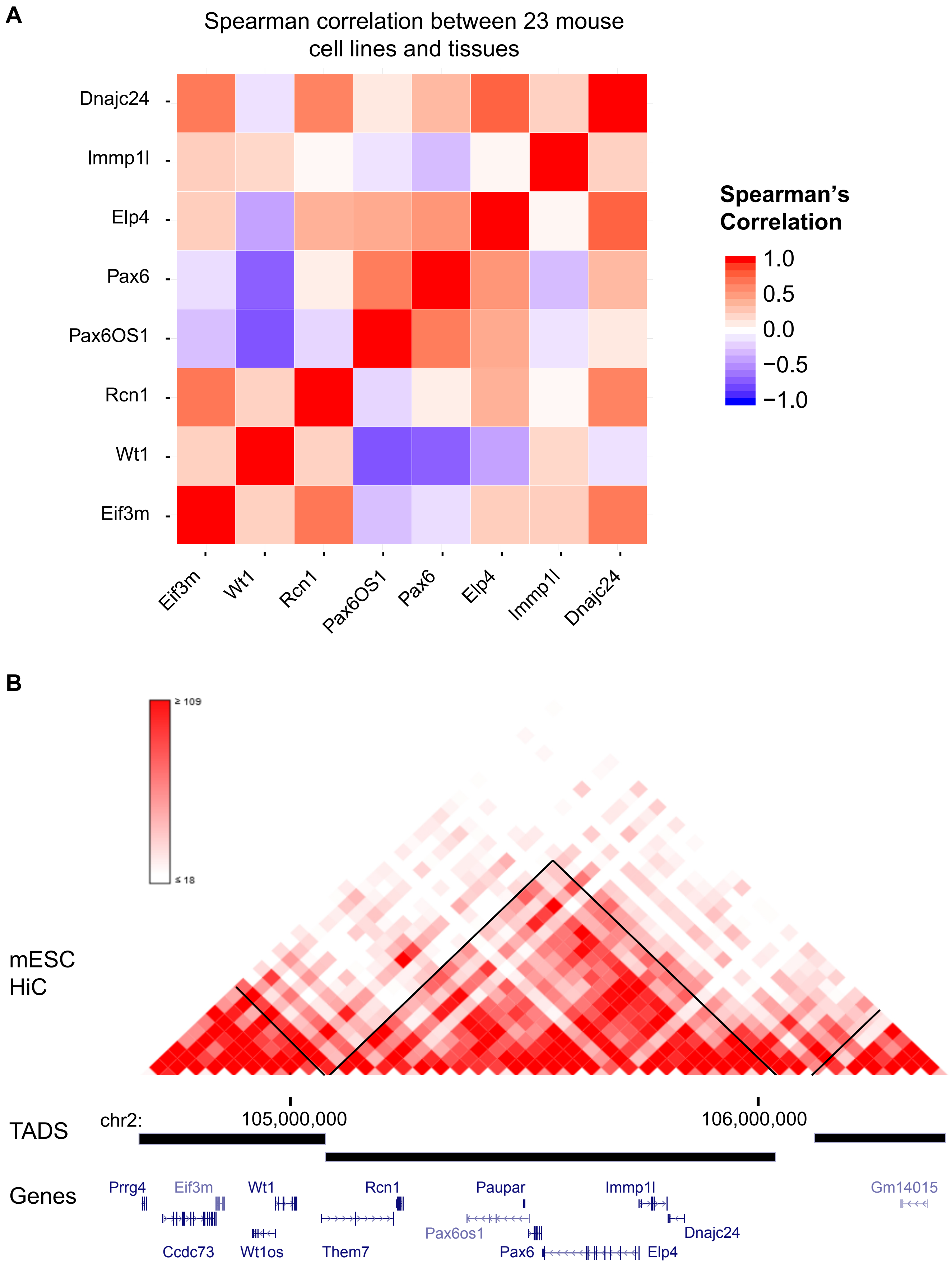

Supplementary Figure 6
